# Peroxidase-Mimicking Activity of Biogenic Gold Nanoparticles Produced from *Prunus nepalensis* Fruit Extract: Characterizations and Application for the Detection of *Mycobacterium bovis*

**DOI:** 10.1021/acsabm.2c00180

**Published:** 2022-05-12

**Authors:** Bhaskar Das, Javier Lou-Franco, Brendan Gilbride, Matthew G. Ellis, Linda D. Stewart, Irene R. Grant, Paramasivan Balasubramanian, Cuong Cao

**Affiliations:** † School of Biological Sciences, Queen’s University of Belfast, Belfast BT9 5DL, U.K.; ‡ Department of Biotechnology and Medical Engineering, National Institute of Technology Rourkela, Rourkela 769008, India; § Nanophotonics Centre, University of Cambridge, Cambridge CB3 0HE, U.K.; ∥ Material and Advanced Technologies for Healthcare, Queen’s University of Belfast, Belfast BT7 1NN, U.K.

**Keywords:** green synthesis, biogenic nanoparticles, peroxidase-mimicking, indirect enzyme-linked immunosorbent assay (iELISA), biosensing, *Mycobacterium bovis*

## Abstract

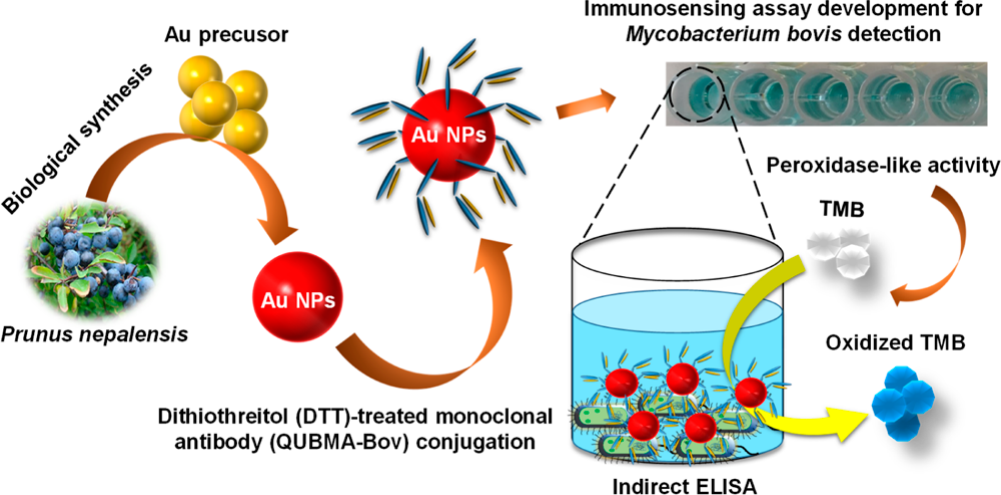

In the present study, a facile, eco-friendly, and controlled synthesis of gold nanoparticles (Au NPs) using *Prunus nepalensis* fruit extract is reported. The biogenically synthesized Au NPs possess ultra-active intrinsic peroxidase-like activity for the oxidation of 3,3′,5,5′-tetramethylbenzidine (TMB) in the presence of H_2_O_2_. Chemical analysis of the fruit extract demonstrated the presence of various bioactive molecules such as amino acids (l-alanine and aspartic acids), organic acids (benzoic acid and citric acid), sugars (arabinose and glucose), phenolic acid, and bioflavonoids (niacin and myo-inositol), which likely attributed to the formation of stable biogenic Au NPs with excellent peroxidase-mimicking activity. In comparison with the natural horseradish peroxidase (HRP) enzyme, the biogenic Au NPs displayed a 9.64 times higher activity with regard to the reaction velocity at 6% (v/v) H_2_O_2_, presenting a higher affinity toward the TMB substrate. The Michaelis–Menten constant (*K*_M_) values for the biogenic Au NPs and HRP were found to be 6.9 × 10^–2^ and 7.9 × 10^–2^ mM, respectively, at the same concentration of 100 pM. To investigate its applicability for biosensing, a monoclonal antibody specific for *Mycobacterium bovis* (QUBMA-Bov) was directly conjugated to the surface of the biogenic Au NPs. The obtained results indicate that the biogenic Au NPs-QUBMA-Bov conjugates are capable of detecting *M. bovis* based on a colorimetric immunosensing method within a lower range of 10^0^ to 10^2^ cfu mL^–1^ with limits of detection of ∼53 and ∼71 cfu mL^–1^ in an artificial buffer solution and in a soft cheese spiked sample, respectively. This strategy demonstrates decent specificity in comparison with those of other bacterial and mycobacterial species. Considering these findings together, this study indicates the potential for the development of a cost-effective biosensing platform with high sensitivity and specificity for the detection of *M. bovis* using antibody-conjugated Au nanozymes.

## 1 Introduction

In recent years, green synthesis of nanomaterials exploiting natural resources is one of the most promising alternatives to the existing physical and chemical methods as this approach does not require high energy consumption, sophisticated instrumentation, and chemical reagents. For example, gold nanoparticles (Au NPs) have been biogenically produced using microorganisms,^1−3^ agricultural waste (e.g., mango peels),^4^ or plant and fruit extracts,^5−10^ demonstrating that the synthesis process is not only rapid, readily scalable, and cost-effective but also eco-friendly and not harmful to biological systems. In addition, the biosynthesized Au NPs have also been reported to possess excellent catalytic activities with improved stability and biocompatibility.^11^ The synthesis mechanism using plant extracts involves the reduction of metal ions due to the presence of natural products comprising functional groups such as phenolic acids, proteins, polyphenol, bioactive alkaloids, terpenoids, and sugars, which play a further role in stabilizing the nanoparticles with improved catalytic efficiency.^12^ Dauthal and Mukhopadhyay demonstrated the biogenic synthesis of stable Au NPs using *Prunus domestica* extracts with excellent catalytic activity for the reduction of 4-nitrophenol.^13^ Furthermore, biogenic Au NPs with excellent peroxidase-mimicking activity have also been reported for various biosensing applications. Kumar et al. demonstrated the size-dependent green synthesis of Au NPs with peroxidase-like activity and subsequent utilization in the development of a colorimetric biosensor for the detection of glutathione from human blood serum.^14^ Similarly, Li and co-workers synthesized stable Au NPs using kiwi extract with higher stability and intrinsic peroxidase-mimicking activity for the colorimetric detection of cysteine.^15^ Additionally, new breakthroughs in biogenic synthesis of nanomaterials have allowed for applications beyond catalysis and biosensing, for example, applications in drug delivery and in bioimaging have been reported.^16^

In this present study, a single-step, facile, and green synthesis of Au NPs at room temperature (RT) using fruit extract of *Prunus nepalensis* (or locally known as “Sohiong”) is presented. *P. nepalensis* has barely been explored among the species within the Rosaceae family, native to different parts of Northeast India. *P. nepalensis* fruit extract possesses a wide range of pharmacological properties as it contains a high concentration of phytochemicals and bioactive compounds and has been used as an astringent, a hepatoprotective agent, or an antioxidant, while the leaves of *P. nepalensis* are also used as a diuretic agent for edema.^17,18^ In this study, we describe the first report of the reducing ability of *P. nepalensis* fruit extract for a cost-effective and eco-friendly one-step biogenic synthesis of Au NPs. The chemical analysis revealed the excellent antioxidant potential of the fruit extract. Further spectroscopic analysis demonstrated the presence of various bioactive molecules such as amino acids (l-alanine, aspartic acids, oxoproline, aminobutanoic acid, and asparagine), organic acids (benzoic acid, malic acid, and citric acid), sugars (arabinose, glucose, and fructose), phenolic acid (protocatechuic acid), saturated fatty acid (palmitic acid), and bioflavonoids (niacin and myo-inositol) in the fruit extract, which potentially acted to enhance the stability and nanozyme activity of the Au NPs. Moreover, the biogenic Au NPs exhibit excellent catalytic efficiency, which is comparable to that of the horseradish peroxidase (HRP) enzyme, a natural enzyme, but with improved environmental stability.^19^ Application of enzyme-mimicking nanoparticles for colorimetric biosensor development has gained popularity as it overcomes the limitations of natural enzymes (i.e., less stability in harsh environmental conditions and cost effectiveness) in the case of biosensor development for point-of-care (POC) applications.^20,21^ However, enhanced catalytic efficiency, cost-effective fabrication, biocompatibility, and high stability remain of paramount concern for effective biosensor development. Over the last few years, *Mycobacterium bovis* is the main causative organism responsible for bovine tuberculosis (bTB) outbreaks, a chronic granulomatous, respiratory disease that affects a cow’s lung tissues and lymph nodes.^22^ The route of transmission to humans occurs predominantly through close contact with the infected animals or consumption of *M. bovis*-contaminated animal products.^23^ To reduce the economic costs of bTB and eliminate the public health risks associated with the consumption of contaminated dairy products, rapid identification of pathogenic mycobacterial species including *M. bovis* is essential for unambiguous diagnosis and an effective disease control strategy.^24^ Thus, there is a need for rapid, cost-effective, user-friendly, and sensitive biosensors for *M. bovis*. Furthermore, in this study, based on the excellent intrinsic peroxidase-mimicking phenomenon, as a proof of concept, the biogenic Au NPs were utilized for the development of a colorimetric approach for the qualitative detection of *M. bovis*. This biosensing strategy could be broadly applied for the development of rapid, cost-effective, and on-site detection of pathogens, biomarkers, or toxins indicating human or animal disease or contamination in food and water.

## 2 Experimental Section

### 2.1 Materials and Reagents

2,2-Di(4-*tert*-octylphenyl)-1-picrylhydrazyl (DPPH), the Tris base (C_4_H_11_NO_3_), hydrochloric acid (HCl), l-ascorbic acid (C_6_H_8_O_6_), phosphate-buffered saline (PBS), trichloroacetic acid (C_2_HCl_3_O_2_), ferric chloride (FeCl_3_), Folin and Ciocalteu’s phenol reagent, sodium carbonate (Na_2_CO_3_), gallic acid (C_7_H_6_O_5_), sodium nitrite (NaNO_2_), aluminum chloride (AlCl_3_), sodium hydroxide (NaOH), catechin (C_15_H_14_O6), hydrogen tetrachloroaurate (III) (HAuCl_4_·3H_2_O), hydrogen peroxide (H_2_O_2_, 30%), the HRP enzyme, 1,4-dithiothreitol (DTT), sodium acetate (NaOAc), and ethylenediaminetetraacetic acid (EDTA) were all purchased from Sigma-Aldrich (UK). 3,3′,5,5′-Tetramethylbenzidine (TMB) was purchased from Thermo Fisher Scientific (UK). PD-10 desalting columns were purchased from GE Healthcare Life Sciences. Solid cheese (cheddar) was purchased from a local supermarket (Tesco, Belfast, UK).

### 2.2 Synthesis of Biogenic Au NPs Using *P. nepalensis* Fruit Extract

*The P. nepalensis* fruit was collected from Meghalaya (25.4670° N, 91.3662° E) in the Northeast region of India. After thoroughly washing and sun-drying, 5 g of dried fruit pulp was cut into small pieces and mixed with 100 mL of distilled water at 60 °C for 10 min under stirring conditions. A solution of *P. nepalensis* fruit extract was prepared by centrifuging at an 8505*g* force for 15 min (Sorvall Legend RT Refrigerated Benchtop Centrifuge, Germany), the resultant solution was filtered using Whatman Grade No. 40 filter paper, and the filtered fruit extract was stored at 4 °C until required (the fruit extract was processed and prepared fresh for every experiment performed during the study). For green synthesis of Au NPs, 80 mL of 1 mM aqueous solution of HAuCl_4_·3H_2_O was added to 20 mL of a 10-fold dilution (in aqueous) of the primary crude extract of *P. nepalensis*. The reaction mixture of the Au salt solution with fruit extract (natural pH of 4.3) was kept at RT overnight under shaking conditions. Optimization of the synthesis process is presented in the Supporting Information.

### 2.3 Optical and Morphological Characterizations of Biogenic Au NPs

Optical analysis of the synthesized Au NPs was performed using a Cary 60 ultraviolet–visible (UV–vis) spectrophotometer (Agilent Technologies, USA). The particle morphology was determined using transmission electron microscopy (TEM-TECNAI TF 30 G2 Super-Twin by FEI, USA) and field emission scanning electron microscopy (FESEM, Nova nanoSEM 450, Czech Republic). The crystal structure of the materials was investigated using the X-ray diffraction (XRD) technique (D/Max 2005, Rigaku, Japan). The surface charge or zeta potential (Zp) of biogenic Au NPs was measured using dynamic light scattering (DLS) techniques (Zetasizer Nano ZS90, Malvern, UK).

### 2.4 Chemical Analysis of *P. nepalensis* Extract

To allow us to postulate the mechanism behind the biogenic synthesis of Au NPs using *P. nepalensis* fruit extract with excellent catalytic efficiency, detailed chemical analysis of the fruit extract was performed. For determining the free radical scavenging activity and reducing ability of *P. nepalensis* extract, several characterization assays were carried out, including (i) a DPPH (2,2-diphenyl-1-picryl-hydrazyl-hydrate) assay, (ii) a reducing power assay, and (iii) total phenolic and (iv) flavonoid content estimation. Detailed description of the free radical scavenging assays is presented in the Supporting Information. To determine the presence of organic bioactive compounds and their different functional groups, *P. nepalensis* fruit extract was characterized using two different analytical spectroscopic techniques. A Fourier transform infrared (FTIR) spectrophotometer (Shimadzu 8201PC, Japan) was used to investigate the presence of the different types of functional groups responsible for the biosynthesis of Au NPs based on the different mode of vibrations. Gas chromatography–mass spectrometry (GC–MS) analysis of the fruit extract was carried out using a GC–MS system (GC: Agilent Technologies 7890N, Palo Alto, California, USA, and MS: quadrupole analyzer, Agilent Technologies 5975C MSD, Palo Alto, California, USA), consisting of a Zebron 7HG-G030-11, ZB-5MSplus capillary column (30 m length, 0.25 mm internal diameter, and 0.25 μm film thickness), with a column temperature range of 0–325 °C (20 °C min^–1^) and an injector temperature of 300 °C.

### 2.5 Characterization of Peroxidase-Mimicking Activity of Biogenic Au NPs

Peroxidase-like activity of biogenic Au NPs was determined using TMB as a chromogenic substrate. Experiments were carried out using a 100 pM concentration of synthesized biogenic Au NPs in a final reaction volume of 1 mL, with 1 mM TMB and 6% (v/v) H_2_O_2_. The reaction mixture was incubated at RT for 10 min, and full-spectrum analysis was carried out using a UV–vis spectrophotometer. Time-dependent UV–vis full spectral analysis of the catalyzed reaction using the same conditions was assessed in a 1 mL final reaction volume for 90 min. To understand the reaction mechanism of TMB oxidation using biogenic Au NPs, 1 mM TMB was catalyzed using the same conditions as those mentioned earlier. The reaction kinetics were monitored at three different wavelengths of 370, 450, and 650 nm for a 20 min time course. Furthermore, to understand the role of *P. nepalensis* fruit extract in the oxidation of chromogenic substrate TMB in the presence of H_2_O_2_, a simple experiment was carried out (see the Supporting Information for more details).

#### 2.5.1 Reaction Kinetics of Peroxidase-Mimicking Activity of Biogenic Au NPs

The reaction kinetics study for the catalytic oxidation of TMB, demonstrating the peroxidase-like activity of biogenic Au NPs, was carried out by recording the absorption values at 370 nm using a microplate reader (Tecan Safire 2, Switzerland). Biogenic Au NPs and HRP were incubated with TMB (0.1–1.0 mM) at various H_2_O_2_ concentrations [0.0075–10% (v/v)] at RT for 10 min. The kinetic parameters were calculated based on the Michaelis–Menten equation, eq 1
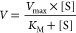
1where *V* is the initial velocity, *V*_max_ is the maximal reaction velocity, [S] is the substrate concentration, and *K*_M_ is the Michaelis constant. For further information on the calculation of parameters, see the Supporting Information. To compare the effect of reaction buffer pH on the catalytic oxidation of TMB, 0.2 M NaOAc buffer solutions with the pH ranging from 2 to 12 were used in the reaction mixtures containing biogenic Au NPs and HRP, respectively, at RT. Similarly, to determine the influence of incubation temperature on catalytic activity, reaction solutions were incubated in a water bath from 20 to 60 °C, while the pH of the reaction mixture was kept constant at 4. The absorption values were measured at 370 nm after 10 min incubation.

### 2.6 Preparation of the Indirect Enzyme-Linked Immunosorbent Assay for the Detection of *M. bovis*

#### 2.6.1 Preparation of Au NPs-QUBMA-Bov Conjugates

For the preparation of biogenic Au NPs-QUBMA-Bov conjugates, thiolation of the *M. bovis*-specific monoclonal IgG antibody (QUBMA-Bov) was performed as previously reported using the DTT reduction method.^25^ The *M. bovis*-specific monoclonal antibody (mAb), QUBMA-Bov, was sourced from a previous study.^26^ In a typical experiment, QUBMA-Bov (0.5 mg) was dissolved in PBS (10 mM, pH 7)/2.5 mM EDTA (500 μL) with a concentration of 1 mg mL^–1^. Ten microliters of 0.5 M DTT was added to PBS (10 mM, pH 7)/2.5 mM EDTA, and the solution was mixed with 500 μL of QUBMA-Bov solution. The reaction mixture was then incubated at RT for 30 min on a rotator.^25^ After the incubation, the mixture was purified through the PD-10 desalting column to remove the excess unutilized DTT molecules. One milliliter of freshly synthesized biogenic Au NPs (4 nM) was centrifuged at 11,200*g* for 30 min, and the pellet was resuspended in PBS (10 mM, pH 7)/2.5 mM EDTA buffer solution. Thiol (SH) group-containing reduced QUBMA-Bov (1 mL) obtained from the DTT reduction method was added to the biogenic Au NP solution (1 mL, 4 nM), and the mixture was incubated on a rotator (RT, 16 h). After the incubation, the mixture was centrifuged at a 7168*g* force for 30 min and resuspended again in 1 mL of distilled water, and this process was repeated twice to remove all the unbound thiolated antibodies. The purified biogenic Au NPs-QUBMA-Bov conjugates were further characterized using a UV–vis spectrophotometer.

#### 2.6.2 Catalytic Efficiency of Au NPs-QUBMA-Bov Conjugates

To determine the peroxidase-mimicking activity of Au NPs-QUBMA-Bov conjugates, the catalytic experiments were carried out using the same reaction conditions as those mentioned in the previous experiments (Section 2.5), and the absorbance of the reaction solution was measured at 370 nm at 1 min intervals for 20 min using a microplate reader (Tecan Safire 2, Switzerland).

#### 2.6.3 Preparation of *M. bovis* Cell Solution

*M. bovis* AF2122/97 was cultured in Middlebrook 7H9 broth consisting of 10% (v/v) oleic acid-albumin dextrose-catalase (both from Difco) (Middlebrook 7H9/OADC broth) until the stationary phase was achieved and then harvested by centrifugation followed by washing in PBS (pH 7.4). To inactivate the cultures, mycobacterial cell suspensions were subjected to a 10 kGy dose of gamma radiation (Gammabeam 650 cobalt irradiator, AFBI, Belfast). After the radiation treatment, samples of each suspension were cultured on Middlebrook 7H10 agar plates and read after 56 days to ensure complete inactivation of all pathogenic species. Irradiated cells were diluted in PBS (pH 7.4) to 10^6^ cfu/mL suspensions and stored at −80 °C.^27^

#### 2.6.4 Indirect Enzyme-Linked Immunosorbent Assay

For the indirect enzyme-linked immunosorbent assay (iELISA), the general procedure is summarized as follows: 96-well microtiter plates (NUNC Maxisorp) were coated with *M. bovis* cells at the final concentration of 10^4^ cfu mL^–1^ in 100 μL of sodium carbonate/bicarbonate buffer (pH 9.6) for 16 h at 4 °C. After the incubation, *M. bovis*-coated wells were washed three times with washing buffer [PBS, pH 7.4, and Tween-20 0.05% (v/v) (PBST)] to remove unbound cells, and the 96-well plate wells were blocked by adding 300 μL/well of blocking buffer [1% (w/v) BSA/PBST] and incubating for 1 h at RT with gentle shaking. After the incubation, wells were washed three times with PBST and kept ready for the assay. For performing the iELISA experiment, *M. bovis* and Au NPs-QUBMA-Bov conjugates were preincubated, as detailed briefly, and standard suspensions containing *M. bovis* cells at concentrations 0, 10, 10^2^, 10^3^, 10^4^, and 10^5^ cfu mL^–1^ were prepared in washing buffer (PBST), and 100 μL of Au NPs-QUBMA-Bov conjugates (diluted 1:5 in PBST) was added to each *M. bovis* cell suspension and incubated for 30 min at RT on a rotator mixer. After 30 min of incubation, 100 μL of a solution containing different concentrations of *M. bovis* and Au NPs-QUBMA-Bov conjugates was added to the respective wells of the microtiter plate and incubated for 30 min at 37 °C at shaking conditions. After the incubation, the wells were washed three times with washing buffer (PBST). Then, 100 μL of the TMB (1 mM) enzyme substrate along with 60 μL of H_2_O_2_ [30% (v/v)] was added to each well and incubated for 15 min at RT on a plate shaker. Color development was stopped by adding 20 μL of the stop solution (2.5 M sulfuric acid). The optical density (OD) was measured at 450 nm using a microplate reader (Tecan Safire 2, Switzerland) within 5 min after stopping the reaction. The absorbance spectra corresponding to each concentration of *M. bovis* were recorded. The OD versus concentration of *M. bovis* calibration curve was obtained by calculating the average reading of three experiments performed under the same conditions. The limit of detection (LOD) of the biosensing strategy was calculated based on the method mentioned by Long and Winefordner, 1983, using the calibration curve data.^28^ The standard deviation value (STDV) for the negative control (NC) was multiplied by 3 (three replicates), and then, it was subtracted from the absorbance value for the NC sample to calculate the *Y* for the lowest detectable concentration. Finally, the *X* (bacteria concentration) in correspondence with the *Y* value was calculated to identify the final LOD concentration. To determine the specificity of the Au NPs-QUBMA-Bov conjugates, the iELISA was performed using four other different bacterial cultures (10^5^ cfu/mL) including irradiated *Mycobacterium smegmatis* mc^2^155 and *Mycobacterium tuberculosis* H37Rv cultures, as well as *Escherichia colik12* ER2738 and *E. coli* BL21 (DE3). Two hundred microliters of washing buffer (PBST) without *M. bovis* was incubated with 100 μL of Au NPs-QUBMA-Bov and used as the NC (blank).

#### 2.6.5 Real Sample Analysis

A homogenized solution of solid cheese (cheddar) was spiked with different concentrations of *M. bovis* (10^0^ to 10^5^ cfu mL^–1^). To prepare the solid cheese matrix, 1*g* of solid cheese was homogenized by using a mortar and pestle in 10 mL of PBST. The cheese solution was then centrifuged at a 664*g* force for 5 min to exclude bigger food particles.^29^ The supernatant was spiked with different concentrations of *M. bovis* cells, and the iELISA was performed as described above.

## 3 Results and Discussion

### 3.1 Synthesis and Characterization of Biogenic Au NPs

#### 3.1.1 UV–Vis Spectrophotometric Analysis

**Figure 1:**
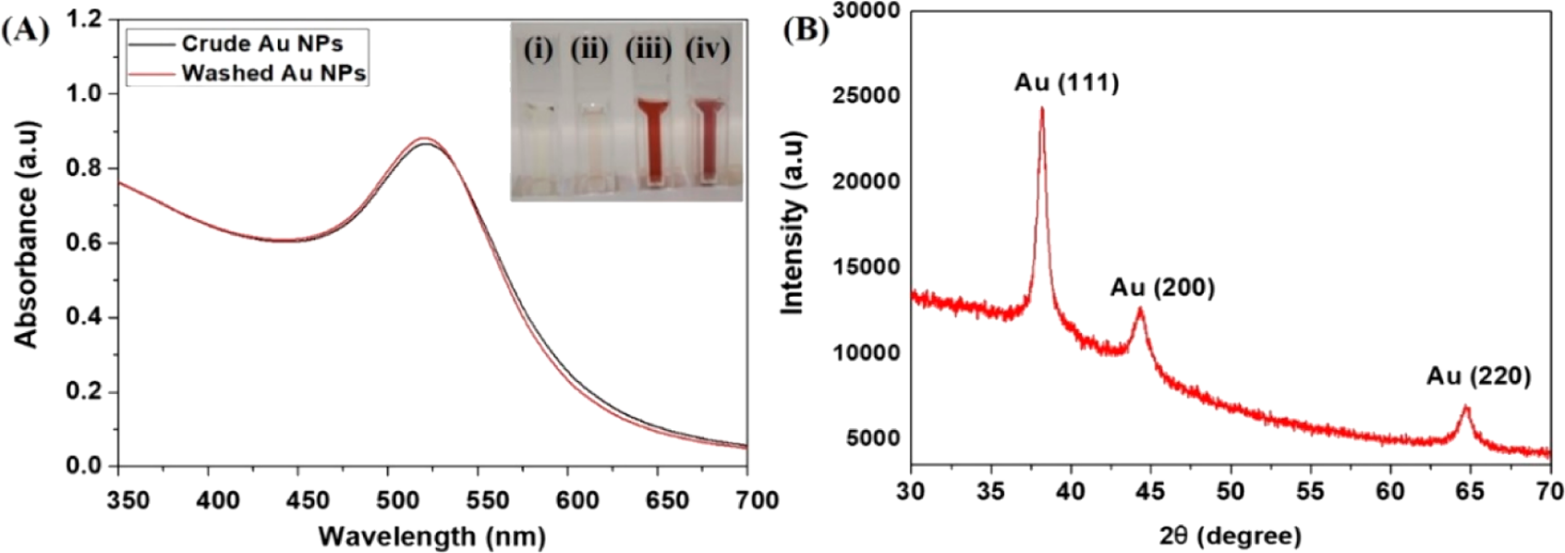
Characterization of biogenic Au NPs. (A) UV–vis spectrum of biologically synthesized Au NPs using *P. nepalensis* fruit extract. The inset shows the color formation of the reaction mixture due to the reduction of the gold salt to form Au NPs: (i) gold salt solution, (ii) *P. nepalensis* fruit extract, (iii) crude Au NPs, and (iv) washed Au NPs. (B) XRD analysis of Au NPs synthesized by *P. nepalensis* fruit extract.

Biosynthesis of Au NPs was followed by the color change in the reaction solution with the appearance of a ruby red color and a change in the surface plasmon resonance (SPR) UV–vis spectrophotometer analysis. The color change occurred after overnight incubation of the Au salt precursor assisted by *P. nepalensis* fruit extract, indicating the formation of Au NPs (Figure 1A inset), attributed to the cumulative oscillation of free surface electrons of metal nanoparticles induced by an interacting electromagnetic field. The absorption spectrum of Au NPs was recorded within the range of 350–700 nm. Figure 1A shows the UV–vis spectrum of unwashed Au NPs recorded with an SPR value at 520 nm, indicating the formation of Au NPs. Further, there was no change in the SPR value after washing steps were carried out, which indicates the stability of the synthesized biogenic Au NPs. The optimized conditions for the Au NP synthesis were found to be the natural pH of the fruit extract (pH 4.3), RT (25–28 °C), a 1 mM precursor salt concentration, and a 10 times (1:10) diluted aqueous fruit extract solution (Figure S1). To investigate the probable synthesis mechanism of Au NPs using *P. nepalensis* fruit extract, chemical characterization of the fruit extract was performed using the total phenolic content measurement, DPPH scavenging activity assay, and reducing power assay, and spectroscopic analysis of the fruit extract was performed using FTIR spectroscopy and GC–MS analysis.

#### 3.1.2 XRD Analysis

The phase and crystal structure of the biologically synthesized Au NPs obtained from *P. nepalensis* extract were investigated using the XRD technique, leading to a better understanding of the atomic positions in its atomic structure. Figure 1B shows the corresponding XRD pattern of the Au NPs in the 2θ range of 20–80°. The XRD spectrum resulted in three intense peaks at 2θ = 38.1, 44.4, and 64.7, which can be indexed as (111), (200), and (220) reflections, respectively, of the fcc (face-centered cubic) structure of metallic gold (JCPDS no. 04-0784).^30^ The absence of any other peak position suggested the formation of monophasic gold nanostructures with pure crystallinity.

#### 3.1.3 TEM Analysis

**Figure 2:**
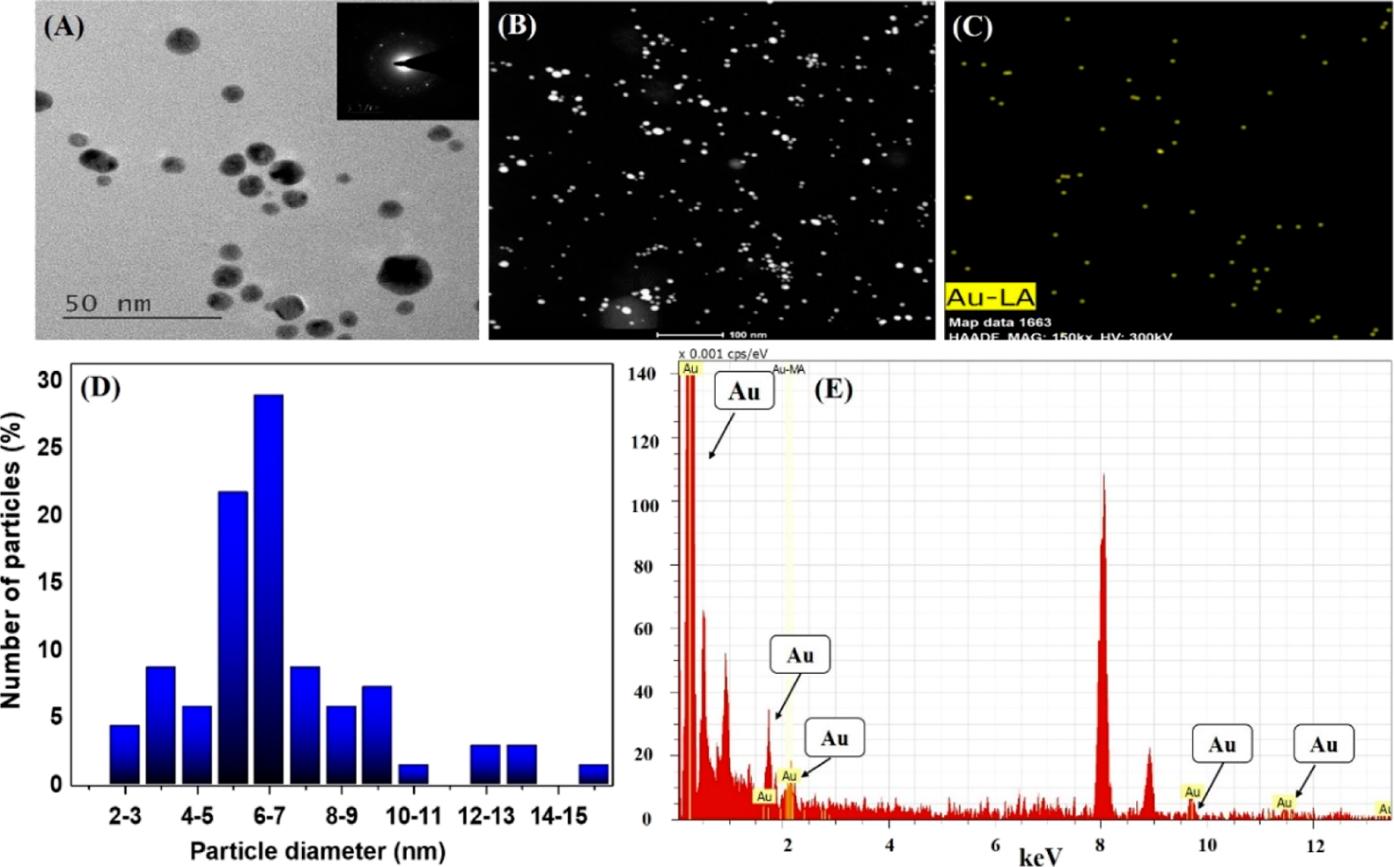
TEM images. (A) Bright field images (inset: SAED pattern). (B) Dark field image. (C) High-angle annular dark field image. (D) Particle size analysis performed on approximately 100 particles using ImageJ software and (E) EDX analysis of biogenic Au NPs synthesized from *P. nepalensis* fruit extract.

Enzyme-mimicking properties of metal nanoparticles strongly depend on their structure and morphology. Figure 2 shows the TEM images of biogenic Au NPs obtained via synthesis using *P. nepalensis* fruit extract, which further confirms the spherical structure of the Au NPs (Figure 2A–C). The size distribution of biogenic Au NPs varies from 2 to 15 nm, with an average particle size of 6 ± 3 nm (Figure 2D). The selected area electron diffraction (SAED) pattern of individual Au NPs shows the lattice pattern structure for the Au NP sample. Most of the plates were truncated and close to the single-crystalline structure corresponding to the fcc structure (Figure 2A inset), which corresponds to the XRD results. TEM energy-dispersive X-ray spectrometry (EDX) was used to understand the chemical composition of the nanoparticles. As shown in Figure 2E, Au has demonstrated a clear peak, clear evidence of the presence of gold (Au) traces in the NP sample with a specific concentration. Furthermore, the FESEM result (Supporting Information, Figure S2) suggested the formation of uniform spherical Au NPs. This observation was in accordance with the data obtained from UV–vis spectrum of Au NPs. The surface charge of the synthesized Au NPs was investigated using zeta potential analysis (Supporting Information, Figure S3), and the surface charge was found to be −20.6 mV, which confirms the presence of a negative charge on the surface of the Au NPs. The negatively charged Au NPs displayed more affinity toward the positively charged TMB substrate for peroxidase-mimicking activity, while also providing the repulsive forces that help prevent Au NPs from aggregating.

### 3.2 Characterization of Peroxidase-Mimicking Activity of Biogenic Au NPs

**Figure 3:**
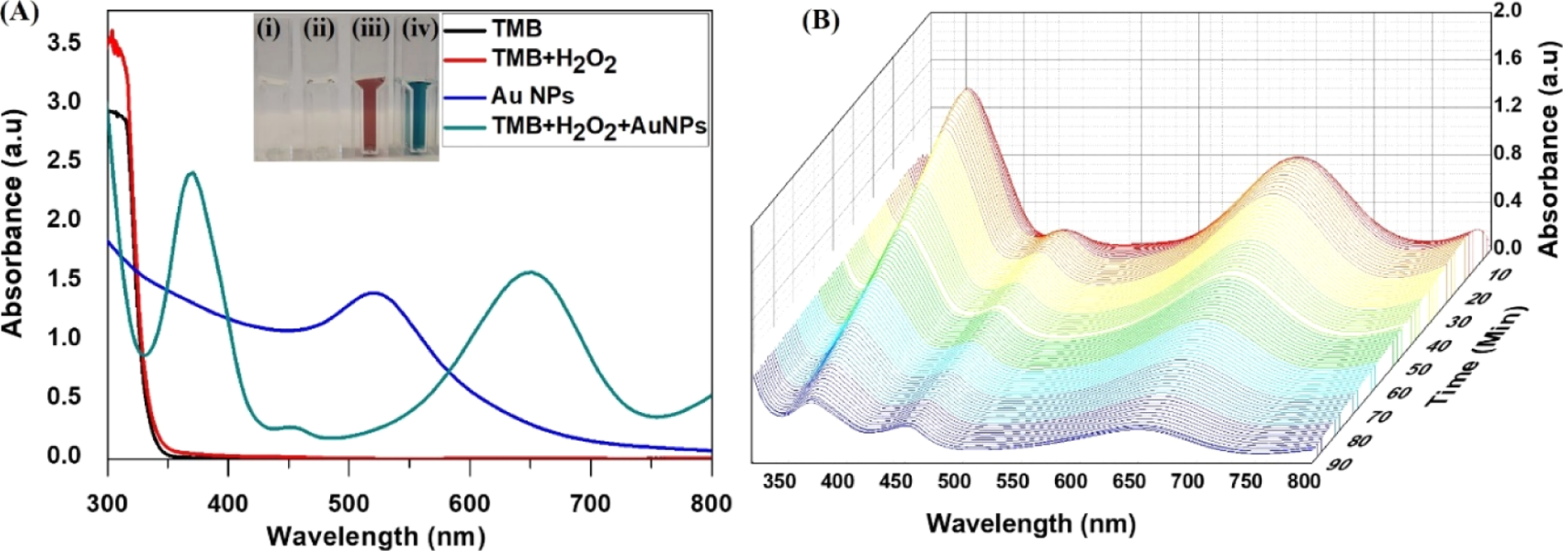
(A) UV–vis spectroscopic analysis of the catalyzed reaction of TMB/H_2_O_2_ in the absence and presence of Au NPs. Figure 3A inset: (i) TMB, (ii) TMB + H_2_O_2_, (iii) Au NPs, and (iv) TMB + H_2_O_2_ +Au NPs. (B) UV–vis full spectral analysis of the catalyzed reaction of 1 mM TMB/6% H_2_O_2_ in the presence of 100 pM Au NPs.

Figure 3 illustrates the evidence of the catalytic activity of biogenic Au NPs, which catalyze the oxidation of TMB in the presence of H_2_O_2_. The Figure 3A inset demonstrates the formation of a blue color in the presence of 1 mM TMB and 6% H_2_O_2_ due to the peroxidase-mimicking activity of biogenic Au NPs. Moreover, the images clearly show that the formation of blue color due to the TMB oxidation requires Au NPs to catalyze the reaction as for the NC samples without Au NPs, (i) only TMB or (ii) TMB with 6% H_2_O_2_, the solution cannot be converted into the blue-colored oxidized TMB form [Figure 3A, insets (i) and (ii)]. Figure 3A showing the full UV–vis spectrum is clear evidence of the TMB oxidation reaction catalyzed by biogenic Au NPs, demonstrated by the formation of charge transfer complexes at different absorption peaks: 370, 450, and 650 nm for biogenic Au NPs in the presence of TMB and H_2_O_2_ (green line).^31,32^ Furthermore, biogenic Au NPs showed their SPR peak at 520 nm (Figure 3A, blue line). However, there is no peak formation found for TMB or TMB with 6% H_2_O_2_, which indicates that no color formation occurred due to the absence of the TMB oxidation reaction without biogenic Au NPs (Figure 3A black and red line). Figure 3B demonstrates the time-dependent UV–vis full spectral scanning kinetic analysis of the TMB oxidation reaction in the presence of biogenic Au NPs along with 1 mM TMB and 6% H_2_O_2_. Within 10 min of the reaction time, the formation of the charge transfer complex shows its highest intensity for the absorption peaks at 370 and 650 nm (Figure 3B). To understand the full reaction mechanism of the TMB oxidation process catalyzed by biogenic Au NPs, a kinetic analysis of the TMB oxidation was performed with 1 mM TMB, 6% (v/v) H_2_O_2_, and 100 pM biogenic Au NPs at set conditions. The reaction kinetics were monitored at three different wavelengths of 370, 450, and 650 nm using a UV–vis spectrophotometer until 20 min (detailed results and discussion in the Supporting Information, Figure S4). The results show an increase in absorbance at 370 nm (Figure S4B) in a time-dependent manner in comparison with that at 450 and 650 nm (Figure S4C,D), while keeping all the reaction parameters similar for every three reactions. Based on this, we proposed to utilize the peak at 370 nm for characterizing the peroxidase-mimicking activity of biogenic Au NPs in kinetic analysis. This result provides significant insights into the peroxidase-mimicking activity of the biogenic Au NPs obtained from *P. nepalensis* extract.

#### 3.2.1 Kinetic Analysis of Peroxidase-Mimicking Activity of Biogenic Au NPs in Comparison with that of HRP

**Figure 4:**
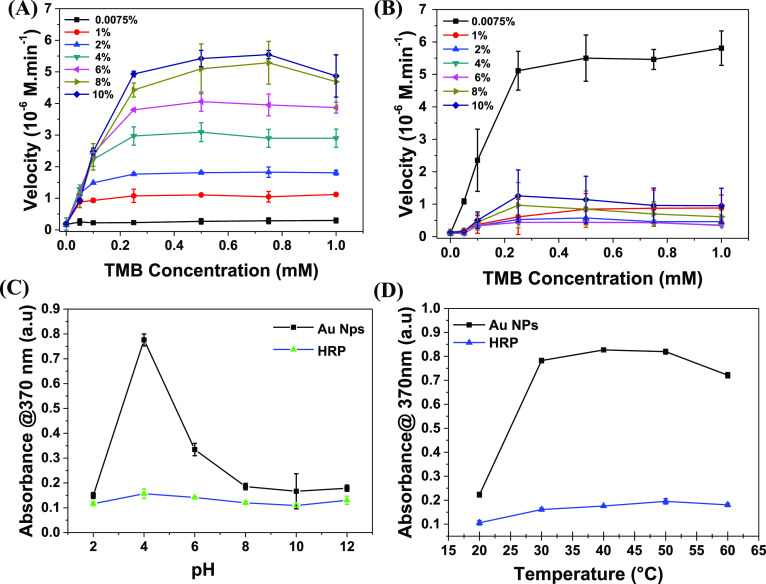
Typical kinetic analysis of TMB oxidation. (A) Kinetic analysis of TMB oxidation by Au NPs in the presence of varying TMB (0–1 mM) and H_2_O_2_ concentrations (1–10%). The concentration of the Au nanozyme was fixed at 100 pM. (B) Kinetic analysis of TMB oxidation by the HRP enzyme in the presence of varying TMB (0–1 mM) and H_2_O_2_ concentrations (1–10%). Samples were analyzed in triplicate (*n* = 3), and the standard deviation was deduced from these data. (C) Kinetic analysis of the HRP enzyme along with Au NPs at different pH values (2–12). (D) Kinetic analysis of the HRP enzyme along with Au NPs at different temperatures (20–60 °C). All the absorbance values were measured at 370 nm.

Kinetic analysis of the TMB oxidation using biogenic Au NPs was performed by mixing various concentrations of TMB/H_2_O_2_ with a fixed concentration of Au NPs and monitoring the oxidation process using absorption analysis at 370 nm. Figure 4A clearly shows the increment in the reaction velocity in accordance with the increased H_2_O_2_ concentration with respect to the TMB concentration in the presence of biogenic Au NPs, which confirms that peroxidase-mimicking activity strongly depends on the H_2_O_2_ concentration (detailed study in Supporting Information, Figure S5). However, in the case of a natural enzyme (i.e., HRP), the reaction velocity decreased significantly with the increasing concentration of H_2_O_2_ from 0.0075 to 10% (Figure 4B). To determine the maximum conversion rate of the substrate to its product by HRP and biogenic Au NPs at the point of the reaction where all the active sites are fully saturated, the maximal velocity (*V*_max_) was calculated. At lower concentrations of H_2_O_2_, such as 0.0075%, the maximum rate of the reaction is found to be 1.68 × 10^–7^ M·min^–1^ for biogenic Au NPs, which significantly increased with the higher concentrations of H_2_O_2_ (10%) up to 5.34 × 10^–6^ M·min^–1^. Further details of the kinetic parameters are summarized in the Supporting Information. From a practical perspective, clinical samples may contain a certain amount of natural peroxidase enzymes, which could potentially interfere with the enzyme-mimicking activity of the Au NPs. Thus, for a better understanding of the kinetic interferences, kinetic analysis of HRP was also performed. Figure 4B clearly shows the catalytic oxidation of TMB using HRP with a maximum reaction velocity of 5.57 × 10^–6^ M·min^–1^ at 0.0075% H_2_O_2_. However, the activity of HRP decreases significantly (5.83 times) when the H_2_O_2_ concentration exceeds 0.0075% (i.e., *V*_max_ = 9.86 × 10^–7^ M·min^–1^ at 1% H_2_O_2_, which decreases accordingly with the increased concentration of H_2_O_2_) (details of the kinetic parameters are summarized in Supporting Information, Table S1). The maximum reaction velocity of TMB oxidation using HRP is much smaller with the increasing concentration of H_2_O_2_ in comparison with that of the biogenic Au NPs. For example, at a moderate level of H_2_O_2_ concentration (i.e., 6% H_2_O_2_), the maximum reaction velocity of biogenic Au NPs is 9.64 times higher than that of HRP (Table S1). The reduced reaction rate is most likely due to the inactivation of HRP at a higher concentration of H_2_O_2_.^33^ Furthermore, this finding can be used to adjust the catalytic response. Therefore, to avoid potential interference from contaminating peroxidase enzymes in clinical samples and to enhance the nanozyme activity of biogenic Au NPs, 6% H_2_O_2_ was chosen for all experiments henceforth. The effect of pH and temperature for the intrinsic peroxidase-like activity of biogenic Au NPs is shown in Figure 4C,D. The results demonstrate that the optimal pH of the reaction solution for biogenic Au NPs is in the range of 3.5–4, which is in agreement with previous reports indicating that in acidic conditions, the intrinsic peroxidase-like activity of biogenic Au NPs increased significantly to catalyze the TMB oxidation (Figure 4C).^34,35^ Moreover, at pH values above the acidic condition (above a pH of 4.0), the catalytic activity decreased, possibly due to the increased redox potential of the substrate, consequently making them less susceptible for oxidation.^36^ Similarly, the effect of temperature on peroxidase-mimicking activity of biogenic Au NPs is shown in Figure 4D. The peroxidase-mimicking activity reaches the maximum at a range of 35–40 °C, which is similar to that of the natural enzyme HRP.^37^

**Figure 5:**
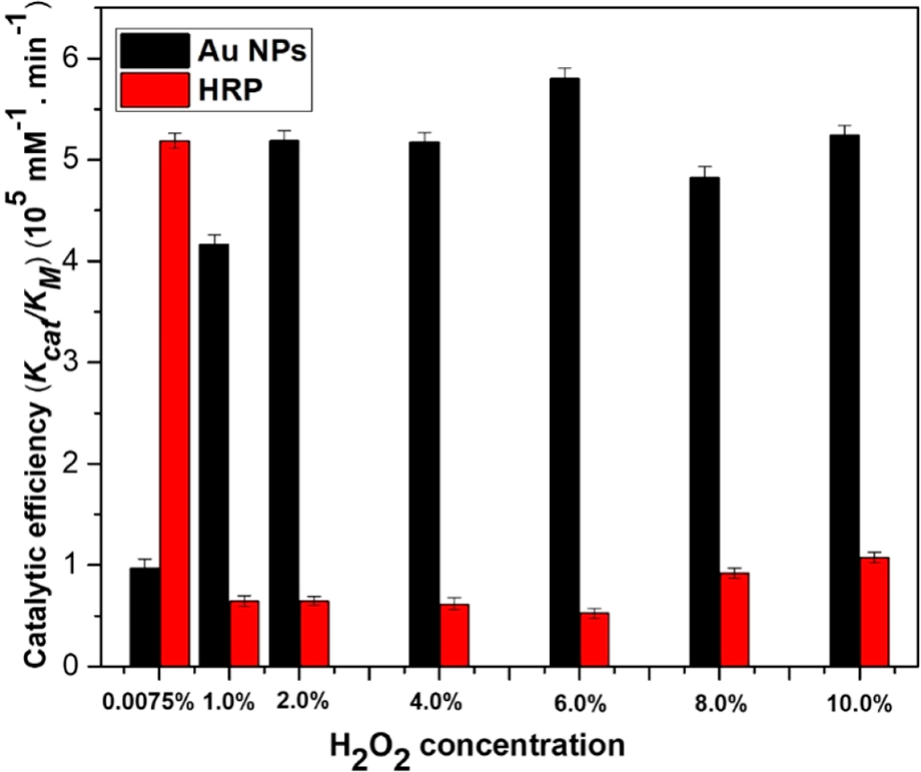
Comparison of the catalytic efficiency of HRP and Au NPs. The reaction was carried out in the presence of 0.1 mM TMB and various H_2_O_2_ concentrations for 10 min at RT. Concentrations of the natural enzyme and nanozyme were fixed at 100 pM, and the absorbance was measured at 370 nm.

To obtain more information on the required substrate concentration for the catalytic reaction, the Michaelis–Menten constant (*K*_M_) was calculated. The results shown in the Supporting Information indicates a lower *K*_M_ value for the biogenic Au NPs in comparison with that for HRP with the increase in the concentration of H_2_O_2_ from 0.0075 to 6%, indicating that less substrate is required to initiate the catalysis reaction at a significant rate with a higher affinity (details of the kinetic parameters are summarized in Supporting Information, Table S1). The *K*_M_ values for both biogenic Au NPs and HRP have changed significantly according to the changes in the H_2_O_2_ concentrations. For instance, from 0.0075 to 6% H_2_O_2_ concentrations, the changes in the *K*_M_ values for Au NPs and HRP are 1.17 × 10^–2^ to 6.9 × 10^–2^ and 1.1 × 10^–1^ to 7.9 × 10^–2^ mM, respectively. The results indicate the higher requirement of the substrate to reach the maximum velocity for HRP-assisted TMB oxidation. For a better understanding of the differences in the reaction rate, the catalytic rate (*k*_cat_) was calculated for both Au NPs and HRP. The results reveal the reduced catalytic rate (*k*_cat_) of HRP with the increasing concentration of H_2_O_2_, that is, the catalytic rate beyond 0.0075% decreased by 5.83 times, whereas in the case of biogenic Au NPs, it increased up to 6.75 times (details of the kinetic parameters are summarized in Supporting Information, Table S1). Figure 5 shows the reduction of the catalytic efficiency (*k*_cat_/*K*_M_) of the HRP enzyme with the increasing concentration of H_2_O_2,_ demonstrating that at 0.0075% H_2_O_2_ (the lowest concentration), the HRP enzyme can convert a higher concentration of the substrate TMB into the product, which further reduced at 6% (v/v) H_2_O_2_. However, for biogenic Au NPs, the catalytic efficiency (*k*_cat_/*K*_M_) was improved up to 6 times at 6% H_2_O_2_, demonstrating the highest conversion rate of the substrate into its product.

### 3.3 Chemical Analysis of *P. nepalensis* Fruit Extract

#### 3.3.1 Free Radical Scavenging Activity and Antioxidant Potential of *P. nepalensis* Fruit Extract

A vast repertoire of secondary metabolites with antioxidant properties found in plant products (e.g., fruit) exhibiting redox capacity have been exploited for biogenic synthesis of nanoparticles.^38^ Estimation of the reducing capacity, antioxidant potential, and radical scavenging ability of plant extracts can be achieved by different electron and hydrogen atom transfer-based assays such as DPPH, Folin–Ciocalteu, and ferric reducing antioxidant power assays.^39,40^ The working principle of these redox reactions is based on the transfer of electrons from the antioxidants present in the plant extract to oxidants such as DPPH or to the metal ions present in the Folin–Ciocalteu reagent. Additionally, this free radical scavenging activity or reducing capacity can be measured by recording the alteration in the absorbance values at specific wavelengths.^41^ Chemical analysis of the *P. nepalensis* fruit extract was performed for a better understanding of the probable role of the bioactive compounds in the biogenic synthesis of Au NPs with higher stability and excellent catalytic efficiency. The basic mechanism behind the green synthesis of metal nanoparticles also relies on the redox reaction where plant or fruit extract with higher total reducing substances are able to produce higher concentration of metal nanoparticles.^41^ Therefore, for *P. nepalensis* fruit extract, the total reducing power assay was performed to determine the electron-donating capability of the biological complex compounds present in the extract, which would act as reducing agents, leading to the formation of Au NPs from its ionic state (i.e., Au^3+^). Table 1 represents the reducing power ability of *P. nepalensis* fruit extract in comparison with that of ascorbic acid as standard. The absorbance value for *P. nepalensis* fruit extract is around 0.447 ± 0.05 (a.u), which is higher than that of ascorbic acid [i.e., 0.300 ± 0.03 (a.u)]. The result indicates the high reduction potential of the fruit extract, which might have resulted in the reduction of Au metal ions to form Au NPs. Similarly, the ability to transfer electrons from antioxidant substances to the unstable free radical molecules due to the antioxidant activity of fruit extracts also plays a significant role in the reduction of metal ions to form metal nanoparticles with excellent catalytic activity. The antioxidant potential of *P. nepalensis* fruit extract was investigated using the DPPH method (517 nm), as shown in Table 1. DPPH acted as a solid free radical, which is reduced by accepting electron or hydrogen from the antioxidant present in the extract. Based on the color changes of the DPPH solution from purple to yellow, the total percent inhibition of the reduced DPPH radical can be measured spectrophotometrically.^42^ The result (Table 1) shows that *P. nepalensis* fruit extract exhibited substantial inhibition of DPPH with 76.091 ± 0.25% RSA (radical scavenging activity) when compared with that of ascorbic acid as a standard (72.22 ± 0.22% inhibition). It is important to note that the antioxidant potential of fruit extract is directly proportional to the quantitative existence of phenolic compounds. Therefore, the higher antioxidant potential of *P. nepalensis* might be due to the presence of phenolic compounds and other secondary metabolites present in the extract, such as anthocyanin and flavonoids.^43^ Total phenolic content in the fruit extract was found to be 43 ± 0.15 μg GAE mL^–1^ (Table 1), which was measured spectrophotometrically at 765 nm by taking gallic acid as the standard. The total flavonoid content of *P. nepalensis* fruit extract was found to be 208.4 ± 0.35 μg CE (catechin equivalent) per mL (Table 1). The antioxidant activity of biological extracts depends on the presence of free OH groups, especially 3-OH groups in flavonoid compounds including flavones, flavonols, and condensed tannins, which are secondary metabolites of plants.^44^ Along with higher polyphenols and flavonoid, *P. nepalensis* is also reported to contain a high amount of anthocyanin.^45,46^ Swer et al. demonstrated the presence of a high amount of anthocyanins (984.40 ± 3.84 mg C3G per 100 g DM), with cyanidin-3-*O*-glucoside, petunidin-3-*O*-glucoside, peonidin-3-*O*-glucoside, and malvidin as the major anthocyanins.^47^

**Table 1:** Free Radical Scavenging Activity and Antioxidant Potential of *P. nepalensis* Fruit Extract

	free radical scavenging activity and antioxidant potential of *P. nepalensis* fruit extract			
sample	DPPH scavenging ability (%)	reducing power (absorbance)	total phenolic content (μg GAE mL^–1^)	total flavonoid content (μg CE mL^–1^)
*P. nepalensis* aqueous fruit extract	76.091 ± 0.25	0.447 ± 0.05	43 ± 0.15	208.4 ± 0.35
ascorbic acid	72.22 ± 0.22	0.300 ± 0.03		

The results of the in vitro antioxidant potential and reducing power assay of the *P. nepalensis* fruit extract suggested that the higher total reducing ability of the fruit extract could be attributed to the presence of phytochemicals with higher antioxidant potentials such as polyphenols and flavonoids. Phenolic compounds present in the *P. nepalensis* such as catechin, caffeic acid, dicaffeoylquinic acid, cinnamic acid, and neo-chlorogenic acid might have assisted the reduction of Au metal ions to form biogenic Au NPs.^46^ For example, Ahmad and Sharma reported the involvement of chlorogenic acid from plant extract in the biogenic synthesis of Ag NPs.^38^ On the other hand, adsorption of different flavonoids and other bioactive compounds over the nanoparticle surface might have acted as the capping and stabilizing agents, which resulted in the formation of stable biogenic Au NPs within a size range of 6 ± 3 nm. Interestingly, the interaction between different phytochemicals present in the *P. nepalensis* extract with the Au metal ions during the synthesis process might have improved the catalytic activity of the Au NPs due to the presence of bioactive functional moieties with high antioxidant properties over the surface of nanoparticles.^13,48^ However, the role that fruit extracts may play in the intrinsic peroxidase-like activity of Au nanoparticles is still unclear. Therefore, as a preliminary attempt to ascertain whether fruit extract can oxidize TMB, a simple reaction was performed, and the results suggested the incapability of fruit extract alone to oxidize TMB (Figure S6, detailed result and discussion in the Supporting Information). Nevertheless, the electrostatic attractions between positively or neutrally charged Au NPs and negatively charged secondary metabolites present in the extract resulted in the formation of a negative surface coating on Au NPs, which further possibly improved the affinity toward positively charged TMB during enzyme-mimicking activity.^43,49^ The presence of various functional groups on the surface of biogenic Au NPs responsible for the improved stability and catalytic activity was investigated by using spectroscopic analysis.

#### 3.3.2 Spectroscopic Analysis of *P. nepalensis* Fruit Extract

**Figure 6:**
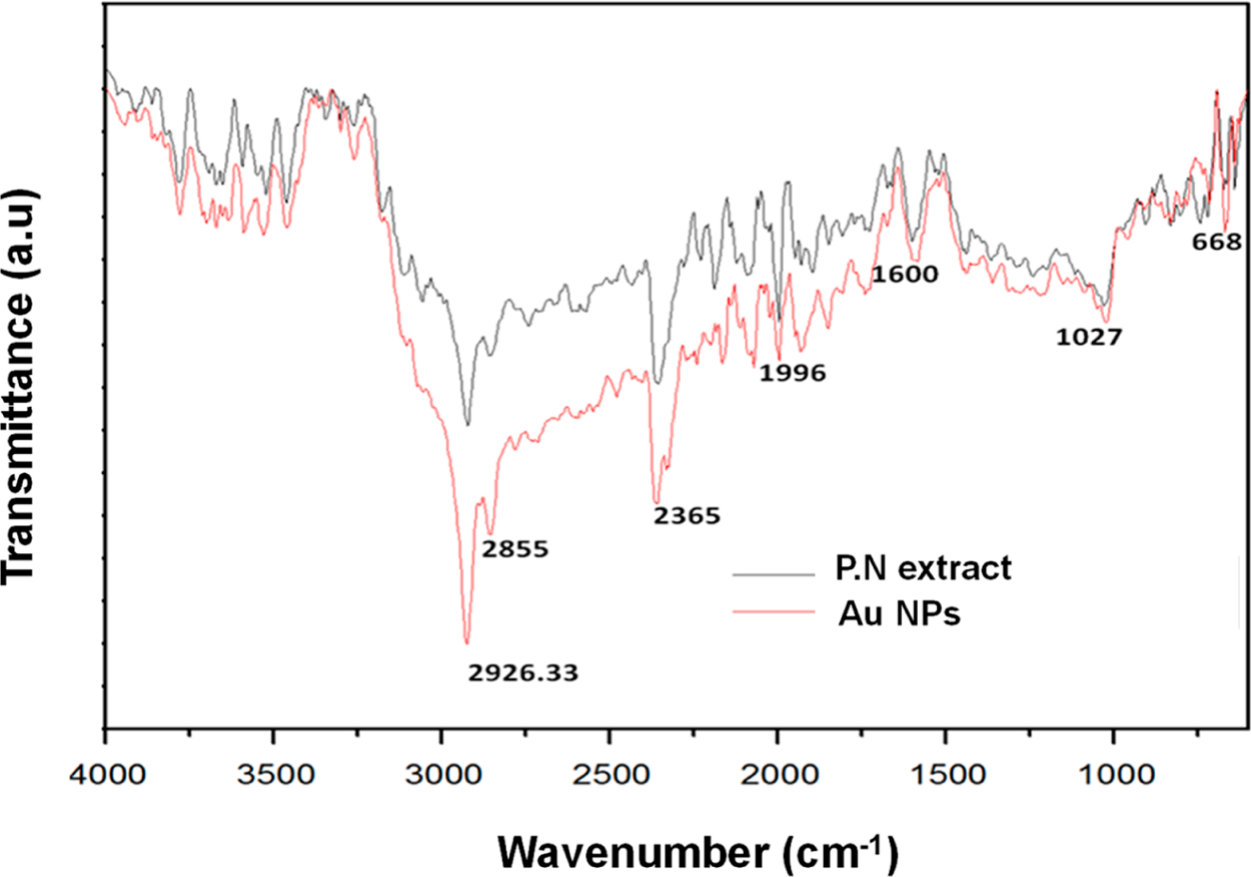
FTIR spectra of biogenic Au NPs synthesized by using *P. nepalensis* extract.

The FTIR spectrum of Au NPs synthesized by using *P. nepalensis* was studied to gain a greater understanding about the possible synthesis mechanism and the presence of functional moieties over the nanoparticle surface (Figure 6). The complex nature of the peaks was expected due to the presence of many biological molecules in the system. There is a slight change in the intensity of the peaks in the two different reaction systems. The presence of a broad peak in the range of 3100–3700 cm^–1^ may correspond to the stretching vibration of NH and OH groups, depicting the traces of phenolic groups.^50^ A strong peak at 2926.3 cm^–1^ in both the spectra represented the C–H stretching of the alkyl group, whereas a slightly intense peak at 2855 cm^–1^ in Au NP spectra indicated the C–H stretching of aldehyde groups.^49^ In addition, there is a strong peak around 2365 cm^–1^ that corresponds to the presence of carboxylic and phenolic groups. A slightly intense peak around 1600–2000 cm^–1^ corresponds to the C=O stretching, corresponding to primary amides and carboxylic groups, and the presence of N–H stretching of amines. Stretched peaks at 1027 and 1029 cm^–1^ represent the presence of the C–N stretching vibration of aliphatic amines in both of the spectra. The peaks around 600 and 800 cm^–1^ are attributed to R-CH groups.^49^ The peak around 668 cm^–1^ in both the spectra confirms the presence of N–H wags from the primary and secondary amines. FTIR analysis of *P. nepalensis* fruit extract and biogenic Au NPs suggested the presence of functional groups associated with different organic bioactive compounds such as polyphenols, enzymes, and proteins in the extract as well as on the surface of biologically synthesized Au NPs. Hence, a simple reaction mechanism can be inferred for synthesizing and stabilizing the Au NPs where bioactive compounds (e.g., phenolic compounds) in the extract possibly acted as a reducing agent. Furthermore, different proteins containing free amino groups, for example, cysteine residues attached on the surface of Au NPs via a thiol linkage, may act as a stabilizing agent. On the other hand, the higher antioxidant potential of phenolic compounds on the surface of Au NPs might have resulted in the enhanced catalytic efficacy of nanoparticles, leading to their excellent intrinsic peroxidase-mimicking activity.^7,9,12,41,49,50^

GC–MS analysis of the *P. nepalensis* fruit extract was carried out to investigate the bioactive organic compounds present in it, as shown in Figure S7. In total, more than 40 compounds were detected and identified (a table containing all the detected and identified compounds is given in the Supporting Information, Table S2) by comparing the obtained mass spectra with the standard mass spectra from the NIST and Wiley electronic libraries supplied with the instrument. Table 2 shows the list of biological compounds present in the fruit extract with higher match factors from GC–MS analysis. Chemical analysis of the fruit extract suggested the presence of various bioactive compounds such as amino acids (l-alanine, aspartic acids, oxoproline, aminobutanoic acid, and asparagine), organic acids (benzoic acid, malic acid, and citric acid), sugars (arabinose, glucose, and fructose), phenolic acid (protocatechuic acid), saturated fatty acid (palmitic acid), and bioflavonoids (niacin and myo-inositol). From these results, it can be postulated that after the reduction of Au metal ions assisted by the polyphenols and other bioactive compounds present in the fruit extract, biogenic Au NPs were further stabilized by these amino acids, sugars, and organic acids due to the capping effect.^50,51^

**Table 2:** List of Biological Compounds Present in the *P. nepalensis* Extract from GC–MS Results

retention time (min)	compound name	formula
5.4558	l-alanine, 2TMS derivative	C_9_H_23_NO_2_Si_2_
6.6430	benzoic Acid, TMS derivative	C_10_H_14_O_2_Si
6.8742	silanol, trimethyl-, phosphate (3:1)	C_9_H_27_O_4_PSi_3_
6.9946	niacin, TBDMS derivative	C_12_H_19_NO_2_Si
8.3407	malic acid, 3TMS derivative	C_13_H_30_O_5_Si_3_
8.5486	l-aspartic acid, 3TMS derivative	C_13_H_31_NO_4_Si_3_
8.5777	l-5-oxoproline, 2TMS derivative	C_11_H_23_NO_3_Si_2_
8.6238	4-aminobutanoic acid, 3TMS derivative	C_13_H_33_NO_2_Si_3_
9.4246	d-arabinose, tetrakis(trimethylsilyl) ether, ethyloxime (isomer 2)	C_19_H_47_NO_5_Si_4_
9.4560	asparagine, 3TMS derivative	C_13_H_32_N_2_O_3_Si_3_
10.2792	protocatechuic acid, 3TMS derivative	C_16_H_30_O_4_Si_3_
10.2950	citric acid, 4TMS derivative	C_18_H_40_O_7_Si_4_
10.3842	d-fructose, 5TMS derivative	C_21_H_52_O_6_Si_5_
11.3433	palmitic acid, TMS derivative	C_19_H_40_O_2_Si
11.7465	myo-inositol, 6TMS derivative	C_24_H_60_O_6_Si_6_

### 3.4 iELISA for the Detection of *M. bovis* Using Biogenic Au NPs

#### 3.4.1 Preparation of Au NPs-QUBMA-Bov Conjugates and Analysis of Peroxidase-Mimicking Activity

**Figure 7:**
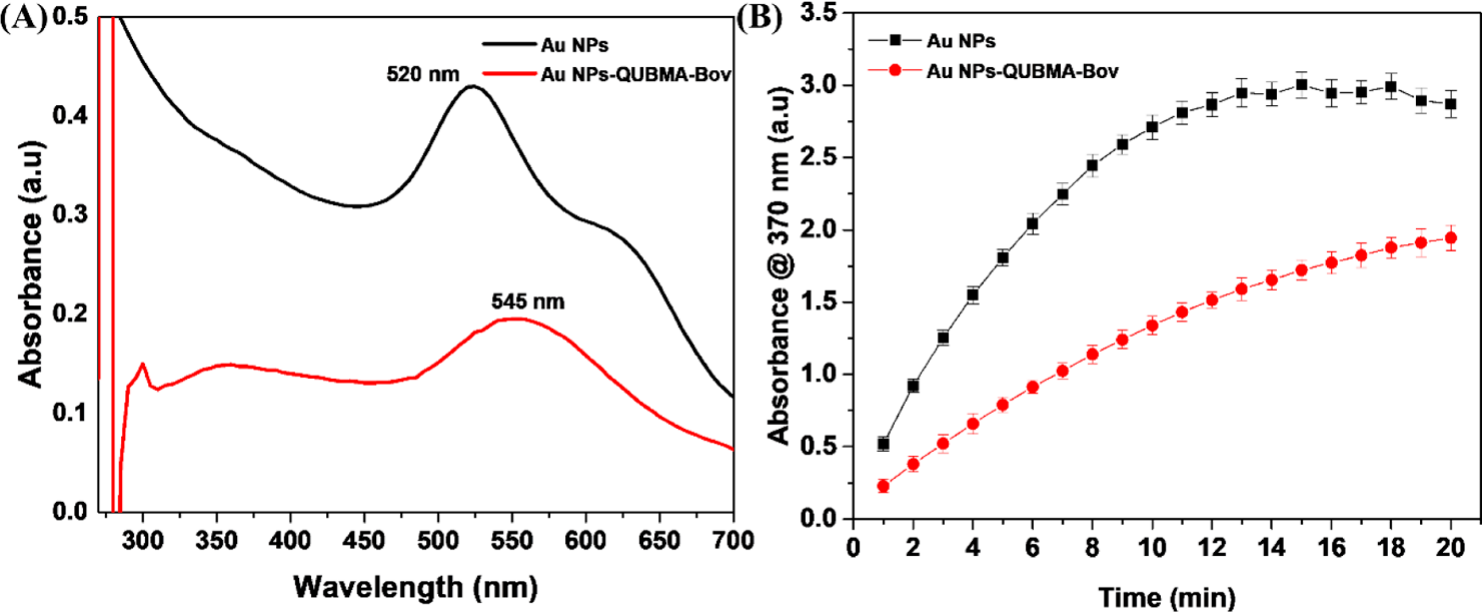
(A) UV–vis spectrum of biogenic Au NPs-QUBMA-Bov conjugates in comparison with that of Au NPs only (B) kinetic analysis of the catalyzed reaction of 1 mM TMB/6% (v/v) H_2_O_2_ in the presence of 100 pM Au NPs and Au NPs-QUBMA-Bov conjugates.

Au NPs-QUBMA-Bov conjugates were prepared by attaching the mAb reduced by DTT onto the surface of biogenic Au NPs. DTT reduces the disulfide bonds, leading to the formation of different fragments containing free cystine residues with thiol (−SH) groups, which readily form covalent bonding with biogenic Au NPs.^52,53^Figure 7A shows the UV–vis spectrum of biogenic Au NPs and Au NPs-QUBMA-Bov conjugates. The result demonstrates a red shift (25 nm) in the localized SPR of Au NPs-QUBMA-Bov at 545 nm with a broadening of the peak, which indicates the formation of Au NPs-QUBMA-Bov conjugates. Additionally, the absorption spectra of Au NPs-QUBMA-Bov conjugates have a sharp characteristic absorption peak near the region of 280 nm, which is indicative of the presence of amino acid residues in the mAb (immuno globular proteins).^54^ Thus, we confirmed the successful conjugation of the antibody on the Au NP surface. To investigate the efficiency of the enzyme-mimicking activity of synthesized Au NPs-QUBMA-Bov conjugates, the catalytic experiment with 0.1 mM TMB in the presence of 6% (v/v) H_2_O_2_ was performed. Figure 7B shows the catalytic efficiency of Au NPs-QUBMA-Bov conjugates in comparison with that of the biogenic Au NPs alone. The result indicates the reduction of the enzyme-mimicking activity of Au NPs-QUBMA-Bov (100 pM) by 32% in comparison with that of biogenic Au NPs after a 20 min reaction time. The reduction in the catalytic efficiency may have occurred due to the interference caused by the antibody coating over the surface of Au NPs during its interaction with TMB. However, the peroxidase-mimicking activity of Au NPs-QUBMA-Bov conjugates can be enhanced by increasing the concentration of Au NPs-QUBMA-Bov for the same substrate concentration (TMB).

#### 3.4.2 iELISA for *M. bovis* Detection

**Figure 8:**
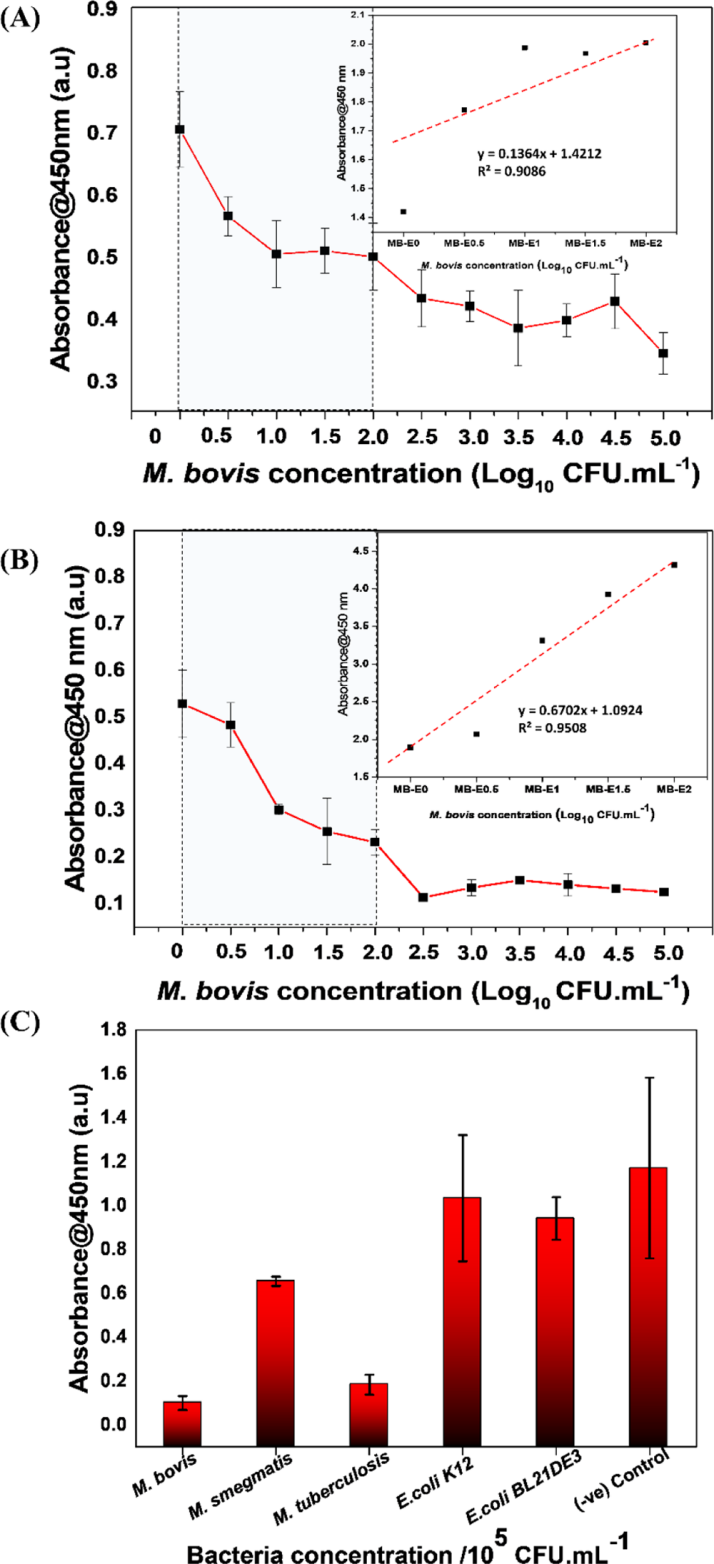
iELISA sensitivity assay using AuNPs-QUBMA-Bov conjugates to detect *M. bovis* (A) in a laboratory buffer solution (PBS, pH 7.4, Tween-20 0.05% v/v, PBST) and (B) in a solid cheese matrix solution. (C) iELISA specificity assay using AuNPs-QUBMA-Bov conjugates to detect *M. bovis* in a laboratory buffer (PBST) solution.

Based on the outcomes of this study, the intrinsic peroxidase-mimicking activity of biogenic Au NPs could be used to create a simple colorimetric biosensor, which can be used possibly in POC settings. Therefore, for the detection of *M. bovis*, an immunoassay was developed using highly peroxidase-mimicking biogenic Au NPs conjugated with a *M. bovis*-specific mAb instead of HRP as a signaling tag. Figure 8A demonstrates the calibration curve for the sensitive detection of *M. bovis* using biogenic Au NPs-QUBMA-Bov conjugates. The result shows the efficiency of biogenic Au NPs-QUBMA-Bov conjugates for a satisfactory level of detection of *M. bovis* within a concentration range of 10^0^ to 10^5^ cfu mL^–1—^, whereas a linear range of detection was found within the range 10^0^ to 10^2^ cfu mL^–1^. Figure 8A clearly shows the decrease in the absorbance value with the increasing concentration of *M. bovis* cells, indicating the presence of free Au NPs-QUBMA-Bov conjugates available after the incubation period to bind with the plate coated with *M. bovis* cells. The higher absorbance response at 10^0^ cfu mL^–1^ indicates the presence of the maximum concentration of free Au NPs-QUBMA-Bov conjugates available to bind with the *M. bovis* cells (coated in the plate). On the other hand, with the increase in the concentration of *M. bovis* cells up to 10^2^ cfu mL^–1^, the absorbance got decreased significantly, indicating the availability of a low concentration of free Au NPs-QUBMA-Bov conjugates for binding as most of the biogenic Au NPs-QUBMA-Bov conjugates have attached with *M. bovis* cells during the incubation period. Interestingly, with the further increase in the *M. bovis* cell concentration (i.e., 10^3^ to 10^5^ cfu mL^–1^), there were no significant changes in the absorbance value. This is probably attributed to the less availability of the active sites on the Au NPs-QUBMA-Bov surface for their substrate TMB to generate a colorimetric signal as most of the active sites could have been preoccupied with the higher concentration of cells during the incubation period (Figure 8A). We further confirmed the linear response of the biosensing strategy by fitting the absorbance value to the concentration of the target analyte up to 10^2^ cfu mL^–1^.^55,56^ The linear regression equation was determined to be *y* = 0.1364*x* + 1.4212*R*^2^ = 0.9086. This study reveals the potential applicability of biogenic Au NPs-QUBMA-Bov conjugates for sensitive detection of *M. bovis* cells within a linear range of 10^0^ to 10^2^ cfu mL^–1^ with an adequate efficiency in the context of previously reported molecular detection techniques. Further, based on the calibration curve data, the LOD was calculated and found to be ∼53 cfu mL^–1^. Stewart and co-workers reported immunomagnetic separation (IMS) along with PCR for the detection of *M. bovis* (from infected animal tissues) with an LOD of 57.7 cfu mL^–1^ within a linear range of 10^0^ to 10^5^ cfu mL^–1^^26^ and *M. bovis* (from infected animals) detection using an IMS-LFD (lateral flow device) with an LOD higher than 10^4–5^ cfu mL^–1^.^27^ In another report, Young et al. developed an RT-PCR assay for *M. bovis* detection with an LOD of 10^4^ cfu mL^–1^ from the soil sample.^57^ However, there are limited reports of commercially available biosensors for the detection of *M. bovis* in contaminated food samples, which can be used in POC diagnosis methods.^58^ A comparative analysis of different biosensing strategies for the detection of *M. bovis* and a few other pathogenic bacteria has been provided in the Supporting Information, Table S3. Our findings could possibly be used for the development of LFD-based POC sensing devices for the rapid and sensitive detection of *M. bovis*. To determine the interference of the experimental matrixes on the detection of *M. bovis* cells using biogenic Au NPs-QUBMA-Bov conjugates, the iELISA was performed in a food matrix (solid cheese) for real sample analysis. Figure 8B shows the absorbance response of the calibration curve for sensitive detection of *M. bovis* cells in the food matrix. The results correspond with the previous findings of a calibration curve for *M. bovis* detection in a laboratory buffer solution. The absorbance response decreases with the increase in the concentration of *M. bovis* cells from 10^0^ to 10^5^ cfu mL^–1^, indicating the moderately sensitive detection capability within the linear range of 10^0^ to 10^2^ cfu mL^–1^ of the analyte. Further, based on the calibration curve data, the LOD was calculated and found to be ∼71 cfu mL^–1^. We further confirmed the linear response of the biosensing strategy by fitting the absorbance value to the concentration of the target analyte up to 10^2^ cfu mL^–1^.^55,56^ The linear regression equation was determined to be *y* = 0.6702*x* + 1.0924, *R*^2^ = 0.9508. However, the absorbance value decreases in comparison with the previous results, probably due to the interference of the food molecules present in the reaction solution, which hinders the oxidation of TMB^31^ catalyzed by biogenic Au NPs-QUBMA-Bov conjugates.

Apart from the sensitivity, specificity is the most essential key influencing factor ensuring the feasibility of the Au NPs-QUBMA-Bov conjugates-based immunosensing assay. To determine the specificity of the biogenic Au NPs-QUBMA-Bov conjugates, the iELISA was performed against four different bacterial strains: (i) two mycobacterial species, *M. smegmatis* mc^2^155 and *M. tuberculosis* H37Rv and (ii) two common *E. colik12* ER2738 and *E. coli* BL21 (DE3) by comparing the absorbance response with each culture isolate. Figure 8C demonstrates that the absorbance responses from *E. colik12*, *E. coliBL21 DE3*, and *M. smegmatis* are similar to the negative signal (only laboratory buffer/PBST without any bacteria), with the higher absorbance attributed to more availability of free unbound biogenic Au NPs-QUBMA-Bov conjugates after the incubation period. Similarly, the absorbance response for *M. bovis* has the lowest absorbance value in comparison to others, indicating that Au NPs-QUBMA-Bov gets attached to the *M. bovis* cells during the incubation period as the mAb conjugated with biogenic Au NPs is specific to *M. bovis*. The absorbance response for *M. tuberculosis* is slightly higher than that of *M. bovis* but less than for the other tested bacteria. These results indicate the specific nature of the mAb toward the *Mycobacterium* species in comparison to the other bacteria tested^27,54^ (Figure 8C).

## 4 Conclusions

In conclusion, an eco-friendly, cost effective, convenient, and single-step green synthesis of biogenic Au NPs was achieved by using *P. nepalensis* fruit extract. To the best of our knowledge, this is the first report for the biogenic synthesis of Au NPs utilizing *P. nepalensis* fruit extract. Chemical analysis of the fruit extract revealed the presence of various bioactive organic compounds such as polyphenols, amino acids, flavonoids, antioxidants, organic acids, and sugars that probably assisted the reduction of Au^3+^ ions followed by the stabilization of the synthesized Au NPs. On the other hand, adsorption of these bioactive compounds over the biogenic Au NP surface not only resulted in them acting as a capping agent but also played an important role in improving the catalytic efficiency of Au NPs. The biogenic Au NPs with a uniform size (6 ± 3 nm) and morphology possess ultra-active intrinsic peroxidase-like activity and can catalyze the oxidation of TMB in the presence of H_2_O_2_. The peroxidase-like activity of biogenic Au NPs followed Michaelis–Menten kinetics, and the reaction velocity was dependent on the environmental pH, temperature, substrate, and H_2_O_2_ concentration. In comparison with the natural enzyme (HRP), biogenic Au NPs showcased a 9.64 times higher maximum reaction velocity at 6% H_2_O_2_ with a higher affinity toward substrate TMB. The Michaelis–Menten constant (*K*_M_) values for biogenic Au NPs and HRP were found to be 6.9 × 10^–2^ and 7.9 × 10^–2^ mM, respectively, at the same nanozyme/enzyme concentration of 100 pM and at 6% H_2_O_2_. Under the experimental conditions, the peroxidase-mimicking activity of biogenic Au NPs was suppressed by 32% after mAb conjugation.

As a proof-of-concept for suitable biosensing applications with biogenic Au NPs, an iELISA was developed to detect *M. bovis* cells using biogenic Au NPs-QUBMA-Bov conjugates, where Au NPs-QUBMA-Bov acted as a peroxidase mimic and a signaling tag. Further experiments were carried out using the iELISA method, and from the absorbance response, it was found that the biogenic Au NPs-QUBMA-Bov conjugates are capable of detection of *M. bovis* within a lower range of 10^0^ to 10^2^ cfu mL^–1^ with decent specificity. These findings suggest that biogenic Au NPs can be used to fabricate an efficient and cost-effective colorimetric immunosensing device that can enable a rapid and reliable qualitative detection of analytes with the naked eye through the use of a peroxidase-mimicking property. Furthermore, this developed biosensing strategy could potentially be integrated into the design of LFD with modifications for in situ testing and could be useful in POC diagnostics.

## Supporting Information Available

Optimization study of the biogenic synthesis process of Au NPs; chemical analysis of *P. nepalensis* extract; free radical scavenging activity and antioxidant potential of *P. nepalensis* fruit extract; reducing power assay; DPPH (2,2-diphenyl-1-picryl-hydrazyl-hydrate) scavenging ability; determination of the total phenolic content; determination of the total flavonoid content; characterization of peroxidase-mimicking activity of biogenic Au NPs; understanding the reaction mechanism of TMB oxidation catalyzed by peroxidase-mimicking biogenic Au NPs; determining the role of *P. nepalensis* fruit extract in the catalysis of TMB oxidation; additional results and discussion: optimization study, FESEM analysis, and characterization of the peroxidase-mimicking activity of biogenic Au NPs; UV–vis spectra of optimization study of Au NP synthesis using *P. nepalensis* extract; FESEM image of Au NPs; zeta potential analysis of the biogenic Au NPs; reaction mechanism of the TMB oxidation process; UV–vis absorption spectra of the reaction kinetics of TMB oxidation; role of *P. nepalensis* fruit extract in the TMB oxidation process; GC–MS analysis of the *P. nepalensis* fruit extract; UV–vis absorption spectra (370 nm) of the reaction kinetics of TMB oxidation comparative analysis; Michaelis–Menten kinetic parameters calculation; GC–MS analysis of *P. nepalensis* extract; and comparison between different biosensing assays for the detection of bacteria (PDF)
