# Supplementary material for: Peroxidase-Mimicking Activity of Biogenic Gold Nanoparticles Produced from Prunus nepalensis Fruit Extract: Characterizations and Application for the Detection of Mycobacterium bovis
Source: ACS Appl Bio Mater. 2022 May 12;5(6):2712–25. doi: 10.1021/acsabm.2c00180 (PMC9214696; doi:10.1021/acsabm.2c00180)
Supplement: Supplementary file 1 — mt2c00180_si_001.pdf [file mt2c00180_si_001.pdf]

Supporting Information:

Peroxidase-mimicking Activity of Biogenic Gold Nanoparticles Produced by *Prunus nepalensis* Fruit Extract: Characterizations and Application for the Detection of *Mycobacterium bovis*

Bhaskar Das[1,2], Javier Lou-Franco[1], Brendan Gilbride[1], Matthew G. Ellis[1,3], Linda D. Stewart[1], Irene R. Grant[1], Paramasivan Balasubramanian[2], Cuong Cao[1,4*]

[1]School of Biological Sciences, Queen's University of Belfast, Belfast

BT9 5DL, United Kingdom

[2]Department of Biotechnology and Medical Engineering, National Institute of Technology Rourkela, Rourkela 769008, India.

[3]Nanophotonics Centre, University of Cambridge, Cambridge CB3 0HE, United Kingdom

[4]Material and Advanced Technologies for Healthcare, Queen's University of Belfast, Belfast BT7 1NN, United Kingdom

*Corresponding author. Email: c.cao@qub.ac.uk

Table of contents:

1. Additional Experimental Details

2. Additional Results and Discussions

2. Additional Figures (Figures S1-S8)

4. Additional Tables (Tables S1-S3)

1. Additional Experimental Details

1.1. Optimization study of biogenic synthesis process of Au nanoparticles

Four parameters were monitored for optimizing the process of Au nanoparticle synthesis: temperature, reaction pH, reductant dilution factor and concentration of gold salt. The process was first optimized for the reaction pH and temperature. Four different reaction pH: 4.0, 7.0, 10.0, and 4.3 (the natural pH of the reductant) were taken and synthesis was observed at five different reaction temperatures:room temperature (RT), 4 °C, 50 °C, 70 °C and 90 °C. Four different concentrations of gold salt solution: 0.5 mM, 1 mM, 2 mM, 3 mM, and 4 mM were used for the optimization study and, similarly, four different dilutions of the reductant were prepared in aqueous solution: 1(undiluted), 1:10, 1:100 and 1:1000. Spectroscopic results were used to analyze each pH and temperature condition and select the optimal parameters for biogenic synthesis of Au NPs.

1.2. Chemical analysis of *Prunus nepalensis* extract

1.2.1. Free radical scavenging activity and antioxidant potential of *P. nepalensis* fruit extract

To understand the chemical characteristics of the *P. nepalensis* fruit extract, and estimate the antioxidant potential and reducing ability, different assays were performed. Likewise, total phenolic content and flavonoid content responsible for the antioxidant and reduction potency were also assessed.

1.2.2. Reducing power assay

Reducing power assay was performed by adapting the protocol described by Yildirim *et al.*[1]. *P. nepalensis* fruit extract (20 µL) was mixed with 2.5 mL of phosphate buffer (0.2 M, pH 6.6) followed by 2.5 mL of potassium ferricyanide as described by Yang et al. [2]. After 30 min incubation at 50 ºC, 2.5 mL of trichloroacetic acid was added and centrifuged at 1008 g force for 10 min [3]. The supernatant (2.5 mL) was mixed properly by pipetting with 2.5 mL of distilled water followed by 0.5 mL of ferric chloride. Finally, the absorbance was measured at 700 nm using a UV-Visible spectrophotometer where the reducing power is directly proportional to the absorbance value [4,5].

1.2.3. DPPH (2,2-diphenyl-1-picryl-hydrazyl-hydrate) scavenging ability

DPPH scavenging ability was determined according to the method reported by Blois (1958) with slight modifications [6]. Two hundred microliters of *P. nepalensis* fruit extract was mixed with 2 mL of 0.1 mM DPPH and 50 mM Tris-HCl buffer (800 µL, pH 7.4). After 30 min incubation at RT, the reduction of DPPH was measured at 517 nm. Tubes without the samples were taken as control, whereas an ascorbic acid (1 mg/mLl) solution was taken as the standard [4,5]. This is represented as % DPPH radical scavenging activity and is calculated using SEq. (1).

$$DPPH\ scavenging\ activity\ (\%) = \frac{Control\ absorbance{-}Sample\ absorbance}{Control\ absorbance} \times 100 \qquad \text{(SEq.1)}$$

1.2.4. Determination of total phenolic content

Total phenolic content was calculated with a spectrophotometer by using Folin-Ciocalteu reagent [7]. *P. nepalensis* fruit extract (2 mL) was mixed with 10 mL of Folin-Ciocalteu diluted reagent (1/10 with double-distilled deionized water). After 2 min incubation, 8 mL of sodium carbonate was added and the reaction solutions were incubated in dark conditions for 2 h at room temperature. The absorbance was measured at 765 nm [4,5]. Gallic acid standard curve was prepared with the concentration range from 50 to 500 µg/mL. Results are articulated in micrograms (µg) of gallic acid equivalent (GAE) per mL of *P. nepalensis* fruit extract [8].

1.2.5. Total flavonoid content

Total flavonoid content was determined by the aluminium chloride colorimetric method [9]. One mL of *P. nepalensis* fruit extract was mixed with 4 mL of distilled water, followed by the addition of 0.3% (v/v) $NaNO_2$ solution. After incubation (5 min), 0.3% $AlCl_3$ was added and the solution incubated for a further 6 min. Two mL of 1 M NaOH solution was added and the final volume of the reaction mixture was prepared to 10 mL by adding distilled water. After incubation (15 min), the absorbance was read at 570 nm. The total flavonoid content present in the solution was calculated from the calibration curve, and the result expressed as mg catechin equivalent per g dry weight [9].

1.3. Characterization of peroxidase mimicking activity of biogenic Au NPs

Peroxidase like activity of biogenic Au NPs was determined using TMB as a chromogenic substrate. Experiments were carried out using 100 pM of biogenic synthesized Au NPs in a

final reaction volume of 1 mL, with 1 mM of TMB and 6% (v/v) H_2O_2. The reaction mixture was incubated at RT for 10 minutes, and full-spectrum analysis was carried out using UV-Visible spectrophotometer. Time-dependent UV-Visible full spectral analysis of the catalyzed reaction using the same conditions was done in 1 mL reaction volume for 90 min [10].

1.3.1. Understanding the reaction mechanism of TMB oxidation catalyzed by peroxidase mimicking biogenic Au NPs

To understand the reaction mechanism of the TMB oxidation using biogenic Au NPs, 1 mM TMB was catalyzed using the same conditions, mentioned earlier (section **1.3**). The reaction kinetics were monitored at three different wavelengths: 370 nm, 450 nm and 650 nm, using a UV-Visible spectrophotometer for 20 min and samples readings were recorded at one-minute intervals [11].

1.3.2. Determining the role of *P. nepalensis* fruit extract for the catalysis of TMB oxidation

To understand the role of *P. nepalensis* fruit extract for the oxidation of chromogenic substrate TMB in the presence of H_2O_2 a simple experiment was carried out by using 10 µL of fruit extract, 1 mM of TMB, 6 % H_2O_2 in a reaction volume of 1 mL. For a comparative study, 100 pM of biogenic Au NPs was taken as a positive control. For blank, only TMB along with H_2O_2 was taken without any NPs or fruit extract [12].

2. Additional Results and Discussions

2.1. Optimization study

The UV-visible readings for the effect of different parameters in the biogenic synthesis of Au nanoparticles can be seen in Figure S1. Figure S1A represents the optimization study of Au nanoparticles synthesis using different pH levels where natural pH was measured as 4.3 (a mixture of gold salt and *prunus* extract). From this figure, it is clearly visible that at natural pH rapid synthesis of Au nanoparticles took place with a SPR maximum at 520 nm. Additionally, an increase in the yield of the NPs is also noticed as compared to pH 4. A further increase in the pH to 7.0 and pH 10 has resulted in a rapid damping in the intensity of the SPR band. This suggests that the formation of Au NPs is favoured under acidic conditions (2 to 7). An increase in the pH to basic condition either has decreased the formation of initial metal nuclei or may have resulted in the agglomeration of the NPs. Figure S1B shows the effect of reaction temperature for the synthesis of Au NPs where increase in the temperature from 4°C to 90°C

has shown a slight variation in the SP band. At RT (room temperature) yield of the synthesized nanoparticles has been considered as highest with an SP maximum at 522 nm. A decrease in the reaction temperature has resulted in the low-intensity SP band, which is possibly due to the formation of less number of nuclei during the reduction process. Figure S1C shows the effect of concentration of the gold salt solution on the synthesis of Au NPs. Reaction with 1 mM gold salt has shown a notable peak with a SPR maximum at 519 nm. It suggests that with an increase in the concentration from 2 mM to 4 mM, SP peak has shown redshift along with a significant amount of damping. The presence of a higher concentration of gold salt may have led to rapid nucleation and simultaneous growth (heterogeneous process), which might have resulted in the formation of particles with different morphology and size. . Figure S1D shows the effect of the rate of dilution of *prunus* extract on the synthesis of Au NPs. Low dilution has resulted in aqueous NP dispersion with a broad SP peak, which centers at 550 nm. Increasing the dilution factor to 1:10 has produced particles with a sharp SP peak at 510 nm. But, further increasing the dilution factor, 1:100 and 1:1000, has not produced any Au NPs; this is probably due to the insufficient amount of reducing agents required for the synthesis of Au NPs.

2.2. Field Emission Scanning Electron Microscope (FESEM) analysis

Morphological characterization of biogenic Au nanoparticles was done by using field emission scanning electron microscope (FESEM). Agglomerated Au nanoparticles with a particles size range from 20-35 nm can be observed in Figure S2. Particles are present in polydisperse form due to the agglomeration (probably occurred during the sample preparation). Uniformity in the size of Au NPs can be observed. This observation was in accordance with the data obtained from UV-visible spectrum of Au nanoparticles.

2.3. Characterization of peroxidase mimicking activity of biogenic Au NPs

To understand the full reaction mechanism of the TMB oxidation process catalyzed by biogenic Au NPs, a kinetic analysis of the TMB oxidation was performed with 1 mM TMB, 6% H_2O_2, and 100 pM biogenic Au NPs at set conditions for 20 min. Figure S4A demonstrates the reaction mechanism of the TMB oxidation process where TMB, parent diamine (TMB^0) gets oxidized by one-electron oxidation to form cation radical (TMB^{+1}) (λ_{max} 370 nm) giving vivid blue color, which is in equilibrium with a meriquinoid complex or charge transfer complex (CTC) (λ_{max} 650 nm) displaying green color. The reaction is further catalyzed by peroxidase mimicking biogenic Au NPs in the presence of H_2O_2 to form diamine oxidation product (TMB^{+2}) or quinodiimine of yellow color (λ_{max} 450 nm) [13]. Figure S4 B-D, demonstrate the

UV-Visible spectral analysis of TMB oxidation at different stages of the TMB oxidation process at three different wavelengths such as 370, 650, and 450 nm. From previous studies, it has been observed that the peak absorbance at 650 nm has been utilized widely as a diagnostic peak to investigate the peroxidase mimicking activity of Au NPs [10,14–16]. However, interference of physicochemical reaction parameters plays an imperative role in inducing aggregation of Au NPs in the high electrolyte reaction medium, attributing a red-shift of the plasmon peak around 600-700 nm. Thus, the absorption peak around 650 nm, representing the formation of oxidized TMB products is potentially less acceptable due to the potential overlapping of peaks generated from the aggregated nanoparticles. Additionally, Figure S4B shows an increase in absorbance at 370 nm in a time-dependent manner in comparison with 450 nm and 650 nm, (Figure S4C, D) while keeping all the reaction parameters similar for every three reactions. Based on this, we proposed to utilize the peak at 370 nm for characterizing the peroxidase mimicking activity of biogenic Au NPs in kinetic analysis. This result offers significant insight into the peroxidase mimicking activity of the biogenic Au NPs obtained by *P. nepalensis* extract.

Kinetic analysis of the TMB oxidation using biogenic Au NPs was performed by mixing various concentrations of TMB/ H_2O_2 with a fixed concentration of Au NPs and monitoring the oxidation process by absorption analysis at 370 nm. Figure S5A-G demonstrate the UV-Visible absorption spectra of TMB oxidation reaction kinetics in presence of different concentrations of H_2O_2. The results indicate the absorbance values of TMB oxidation at 370 nm increased with the increasing concentrations of TMB and H_2O_2 at fixed concentration of biogenic Au NPs (100 pM). At a lower concentration of H_2O_2 (0.0075 %) the TMB oxidation (for TMB concentration 0-1 mM) catalyzed by biogenic Au NPs occurred at a slower rate generating less amount of oxidized TMB product with the increasing time. However, when the H_2O_2 concentration was increased above 4 % (v/v), the rate of TMB oxidation catalytic reaction increased significantly with respect to time at a fixed concentration of Au NPs (Figure S5D-G). This findings attributed to the availability of the excess amount of the H_2O_2 for accepting the lone pair of electron from the Au NPs surface following with the oxidation of TMB[17].

Biogenic Au NPs showed excellent intrinsic peroxidase mimicking activity in presence of H_2O_2 indicating the ability to oxidize TMB with higher efficiency. For biological synthesis process, *P. nepalensis* fruit extract acted as a reducing as well as a capping agent for the synthesis of stable Au NPs, indicating the probable presence of biological components from

the fruit extract over the surface of the nanoparticles. Hence, the role for fruit extracts in possessing intrinsic peroxidase-like activity of Au NPs remains unknown. As a preliminary test of the ability of fruit extract to oxidize TMB, a simple reaction was carried out with the similar protocol outlined above. Figure S6 shows the bar diagram of the absorbance values at 370 nm for the comparative TMB oxidation efficiency of fruit extract and biogenic Au NPs at different time intervals. The result indicates that within 5 minutes of the reaction, the biogenic Au NPs showed the highest absorbance value at 370 nm indicating the oxidation of TMB, which generated blue color (Figure S6 inset (a&b (iii)) whereas no change in the absorbance value was observed in case of fruit extract. Furthermore, there was no changes in the color of the reaction solution for fruit extract in presence of 0.1 mM TMB and 6 % H_2O_2 attributed to the incapability of fruit extract alone to oxidize TMB (Figure S6 inset (a&b (ii)).[12].

3. Additional Figures

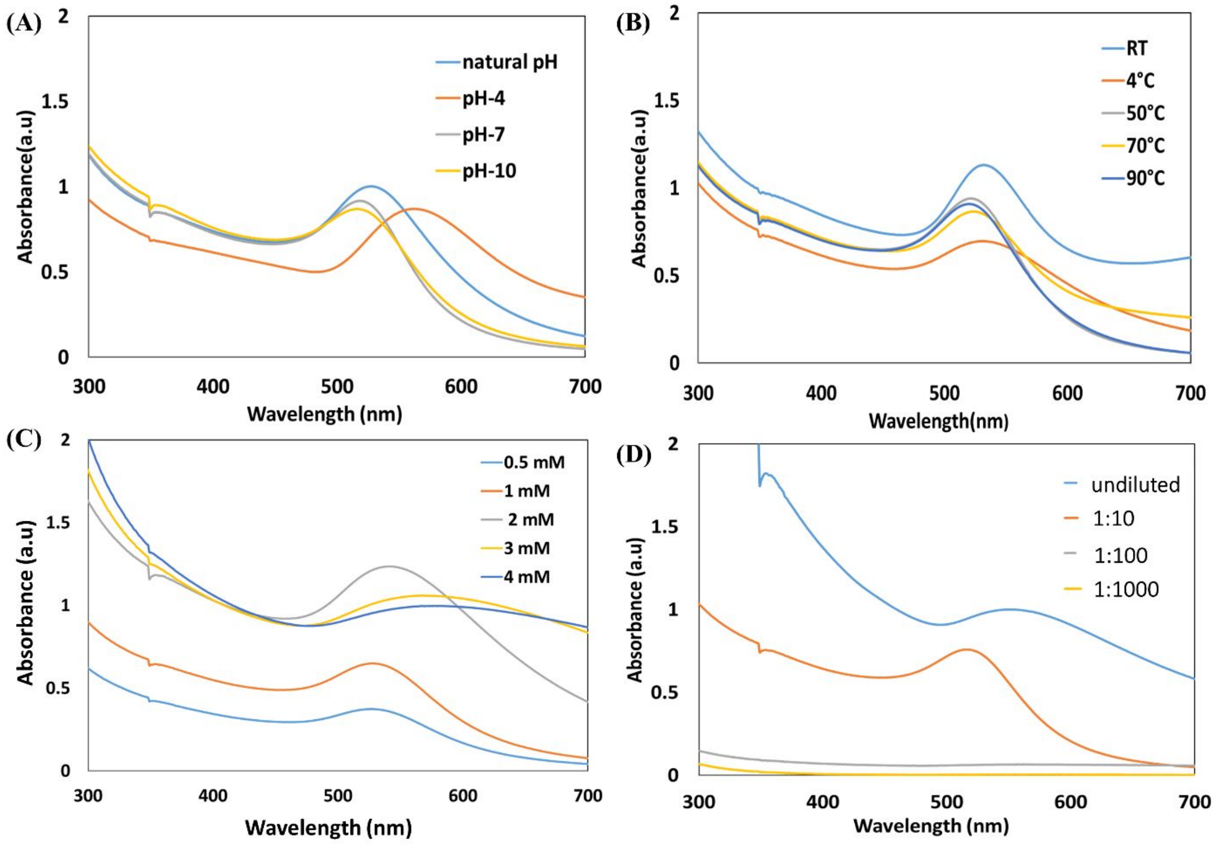

Figure S1. UV-visible spectra of optimization study of Au NPs synthesis using *Prunus nepalensis* extract. (a) Effect of different pH, (b) effect of varying temperature, (c) effect of varying salt concentration, (d) effect of different dilution rates of *Prunus nepalensis* extract.

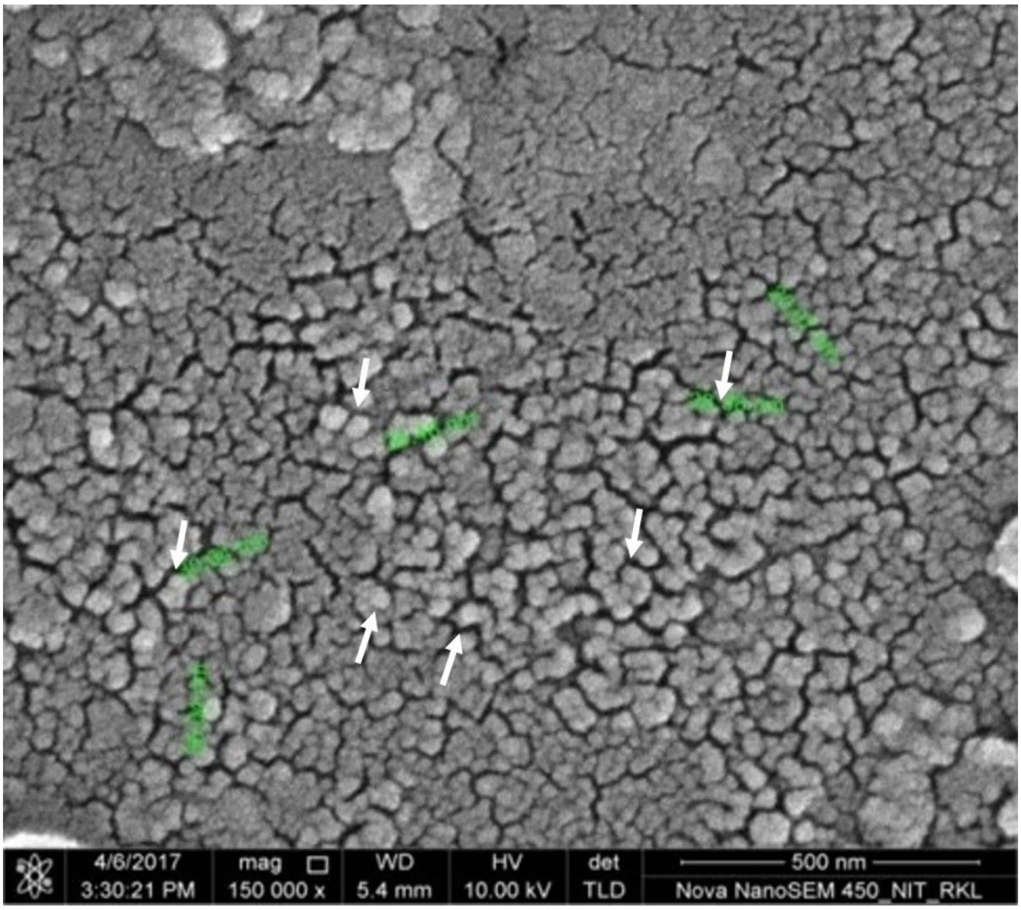

Figure S2. Field emission electron microscopy (FESEM) image of Au NPs synthesized by *P. nepalensis* fruit extract. Arrows indicating the biogenic Au NPs.

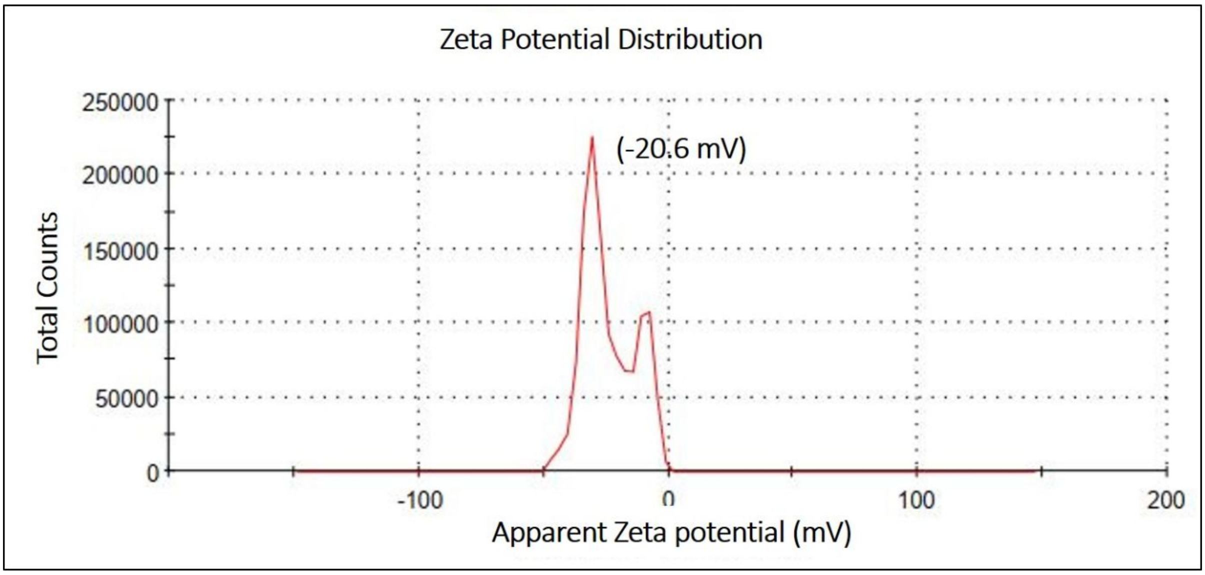

Figure S3. Zeta potential analysis of the biogenic Au NPs synthesized using *Prunus nepalensis* extract.

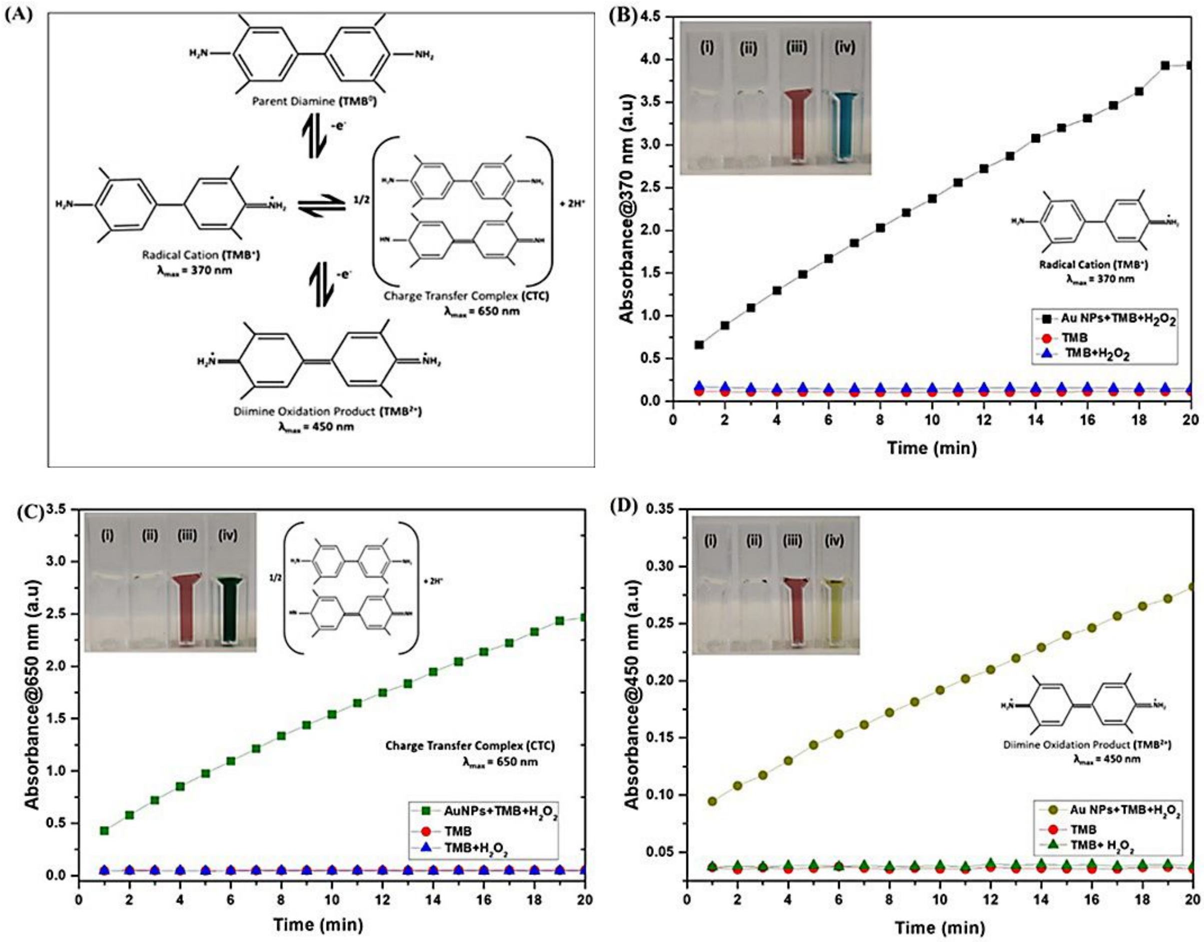

Figure S4. (a) Reaction mechanism of TMB oxidation process. Kinetic analysis of the catalyzed reaction of 1 mM TMB /6% H_2O_2 in presence of 100 pM Au NPs at different wavelengths 370nm (b), 650 nm (c) and 450nm (d) to demonstrate the reaction mechanism of TMB oxidation process (Figure 4. B,C,D Insite: (i) TMB, (ii) TMB+ H_2O_2, (iii) Au NPs, (iv) TMB+ H_2O_2 +Au NPs)

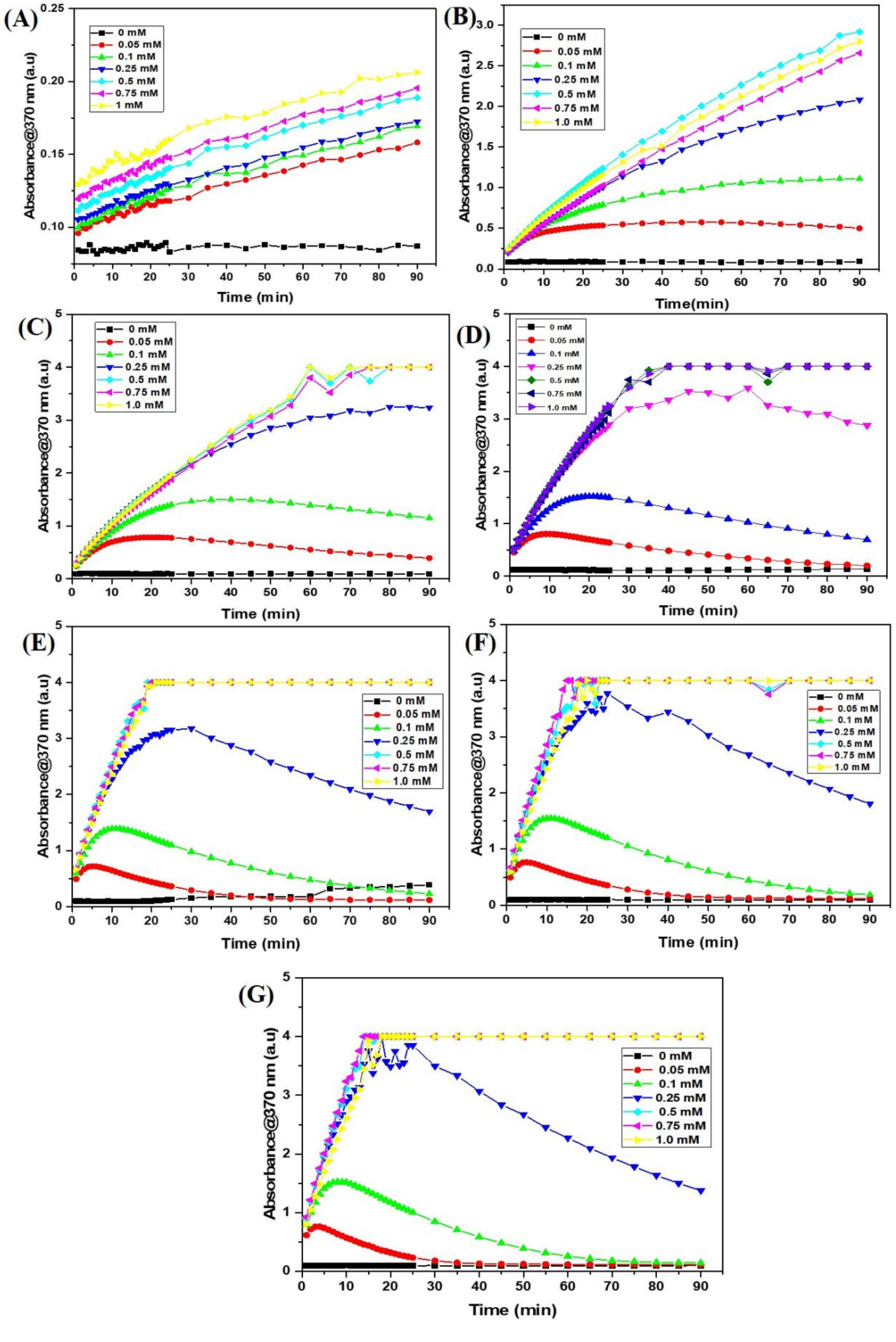

Figure S5. UV-Visible absorption spectra (370 nm) of reaction kinetics of TMB oxidation process varying the concentration of H_2O_2 (a) 0.0075%, (b) 1%, (c) 2%, (d) 4%, (e) 6%, (f) 8%, (g) 10%. The concentration of biogenic Au NPs was kept constant (100 pM) while

different concentrations of TMB (0-1 mM) were utilized to analyse the reaction kinetics up to 90 min of reaction time.

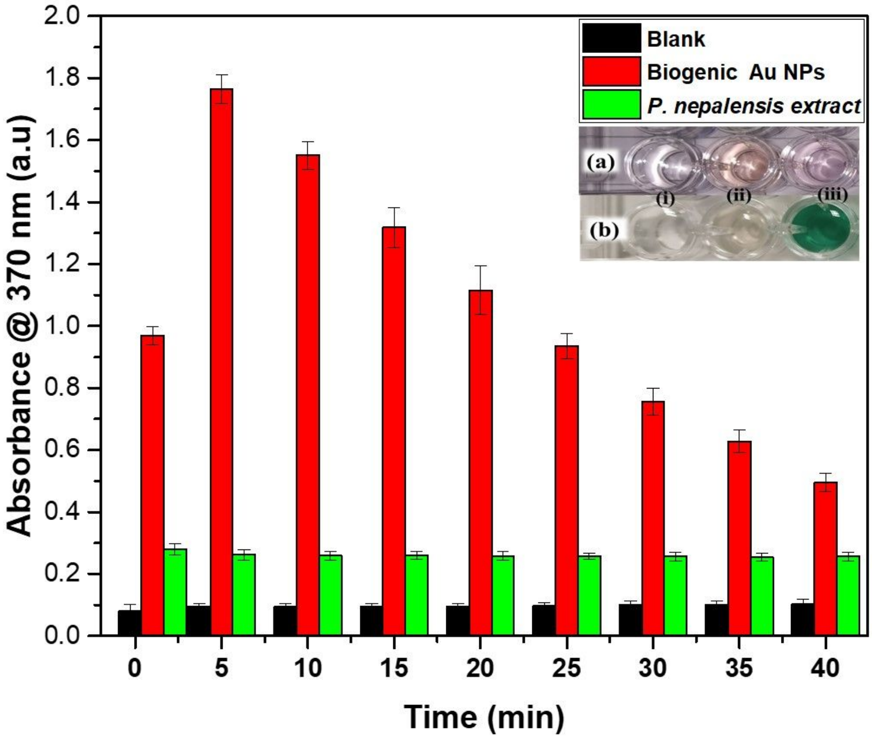

Figure S6. Role of *P. nepalensis* fruit extract in TMB oxidation process. Absorbance values of TMB oxidation process in presence of 1 mM TMB, 6% H_2O_2 with *P. nepalensis* fruit extract (green), biogenic Au NPs (red) and blank (without biogenic Au NPs and fruit extract). (Figure inset visualization of the TMB oxidation reaction at different time intervals 0 min (a), after 10 min (b), with (i) Blank, (ii) fruit extract, (iii) biogenic Au NPs). Samples were analysed in triplicates (n=3) and standard deviation deduced form these data.

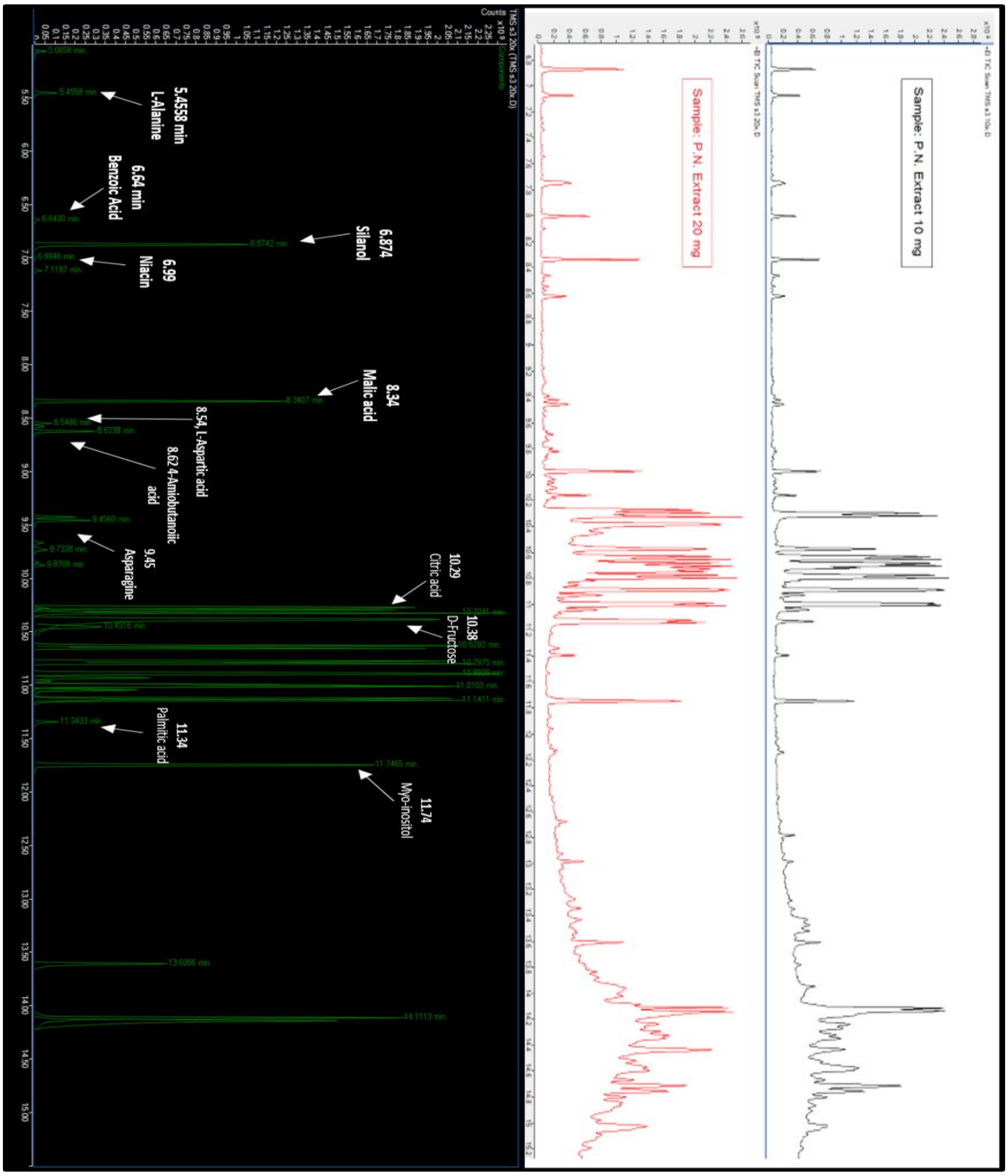

Figure S7. GC-MS analysis of the *P. nepalensis* fruit extract.

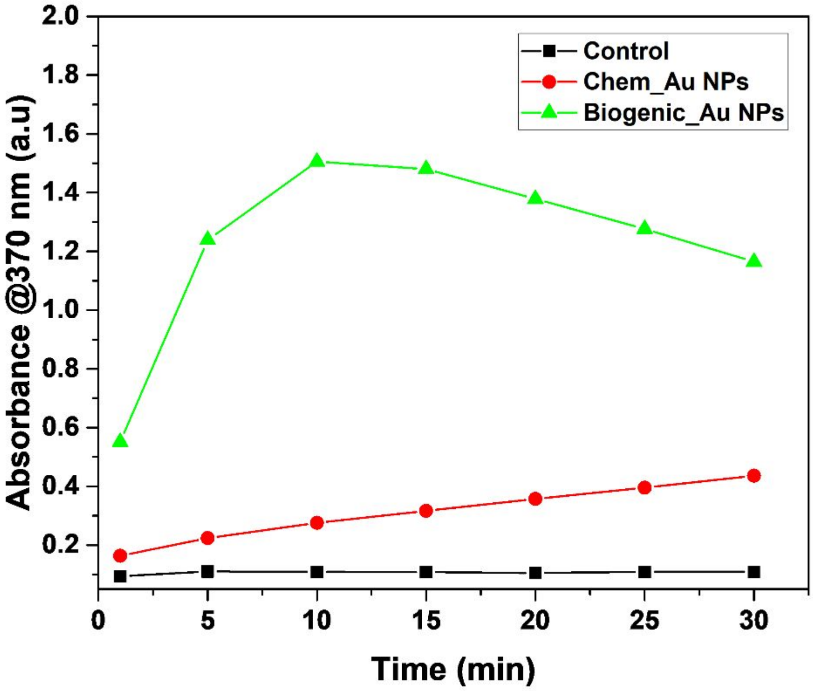

Figure S8. UV-Visible absorption spectra (370 nm) of reaction kinetics of TMB oxidation process using biogenic Au NPs and chemically synthesized Au NPs. The concentration of biogenic Au NPs and chemically synthesized Au NPs was kept constant (100 pM) while 0.1 mM TMB and 6% H_2O_2 was utilized to analyse the reaction kinetics up to 30 min of reaction time.

4. Additional Tables

Table S1. Michaelis-Menten kinetic parameters demonstrating the peroxidase-mimicking activities of HRP enzyme, biogenic Au NPs at different concentrations of H_2O_2. K_{cat} was demonstrated as the ratio of V_{max} to molar concentration of enzyme. The concentration of biogenic Au NPs was calculated according to the previously published article[1]. The concentration of biogenic Au NPs was determined to be 100 pM.

H_2O_2 concentration	Catalyst	V_{max} (M min^{-1})	K_M (mM)	K_{cat} (min^{-1})	K_{cat}/K_M (mM^{-1} min^{-1})
0.0075%	HRP	5.75×10^{-6}	1.1×10^{-1}	5.75×10^{4}	5.19×10^{5}
	Biogenic Au NPs	1.68×10^{-7}	1.17×10^{-2}	1.68×10^{3}	9.69×10^{4}
1.0%	HRP	9.86×10^{-7}	1.5×10^{-1}	9.86×10^{3}	6.44×10^{4}
	Biogenic Au NPs	1.13×10^{-6}	1.12×10^{-2}	1.14×10^{4}	4.16×10^{5}
2.0%	HRP	5.086×10^{-7}	7.8×10^{-2}	5.09×10^{3}	6.47×10^{4}
	Biogenic Au NPs	1.84×10^{-6}	3.5×10^{-2}	1.84×10^{4}	5.19×10^{5}
4.0%	HRP	4.13×10^{-7}	6.7×10^{-2}	4.41×10^{3}	6.17×10^{4}
	Biogenic Au NPs	2.9×10^{-6}	5.7×10^{-2}	3.0×10^{4}	5.17×10^{5}
6.0%	HRP	4.16×10^{-7}	7.9×10^{-2}	4.17×10^{3}	5.24×10^{4}
	Biogenic Au NPs	4.016×10^{-6}	6.9×10^{-2}	4.02×10^{4}	5.80×10^{5}
8.0%	HRP	7.78×10^{-7}	8.4×10^{-2}	7.79×10^{3}	9.21×10^{4}
	Biogenic Au NPs	5.11×10^{-6}	1.06×10^{-1}	5.12×10^{4}	4.82×10^{5}
10.0%	HRP	1.07×10^{-6}	1.00×10^{-1}	1.08×10^{4}	1.08×10^{5}
	Biogenic Au NPs	5.34×10^{-6}	1.02×10^{-1}	5.35×10^{4}	5.24×10^{5}

Table. S2. GC MS analysis of *P. nepalensis* extract.

Retention Time (mins)	Compound Name	Match Factor	Best Hit	Formula	Base peak	Base Peak Height	Base Peak MZ
5.0657	D-(-)-Lactic acid, 2TMS derivative	88.5	True	$C_9H_{22}O_3Si_2$	31832558.7	23075921.5	146.8
5.0658	Lactic Acid, 2TMS derivative	85.4	True	$C_9H_{22}O_3Si_2$	30486708.9	22447359.1	147.0
5.4558	L-Alanine, 2TMS derivative	96.5	True	$C_9H_{23}NO_2Si_2$	37169626.6	39654803.8	115.9
6.6430	Benzoic Acid, TMS derivative	92.7	True	$C_{10}H_{14}O_2Si$	5868425.7	6980443.0	179.0
6.8742	Silanol, trimethyl-, phosphate (3:1)	92.6	True	$C_9H_{27}O_4PSi_3$	151859382.8	111219864.8	299.0
6.9946	Niacin, TBDMS derivative	92.5	True	$C_{12}H_{19}NO_2Si$	912420.4	882556.4	179.8
7.1187	Butanedioic acid, 2TMS derivative	85.3	True	$C_{10}H_{22}O_4Si_2$	14771708.6	16226669.6	147.0
8.3407	Malic acid, 3TMS derivative	97.8	True	$C_{13}H_{30}O_5Si_3$	121527394.2	107029973.7	72.9
8.5486	L-Aspartic acid, 3TMS derivative	94.5	True	$C_{13}H_{31}NO_4Si_3$	15638448.1	18859400.3	232.0
8.5777	L-5-Oxoproline, , 2TMS derivative	92.4	True	$C_{11}H_{23}NO_3Si_2$	18332164.4	19450196.0	155.9
8.6238	4-Aminobutanoic acid, 3TMS derivative	95.8	True	$C_{13}H_{33}NO_2Si_3$	58110907.6	59860974.8	173.9
9.4246	D-Arabinose, tetrakis(trimethylsilyl) ether, ethyloxime (isomer 2)	95.8	True	$C_{19}H_{47}NO_5Si_4$	29455211.9	31603140.3	72.9
9.4560	Asparagine, 3TMS derivative	97.0	True	$C_{13}H_{32}N_2O_3Si_3$	51602786.1	42892177.3	72.9
9.6669	Isocitric acid lactone, 2TMS derivative	89.2	True	$C_{12}H_{22}O_6Si_2$	8127325.9	9204626.4	72.9
9.7338	D-Ribose, 4TMS derivative	89.3	True	$C_{17}H_{42}O_5Si_4$	17240072.0	10547564.4	72.9
9.8496	Aconitic acid, (Z)-, 3TMS derivative	85.1	True	$C_{15}H_{30}O_6Si_3$	6264977.2	6639073.9	146.8
9.8769	D-(+)-Ribono-1,4-lactone (R,S,R)-, 3TMS derivative	86.1	True	$C_{14}H_{32}O_5Si_3$	11466409.4	11196669.3	72.9
10.2717	D-(-)-Fructofuranose, pentakis(trimethylsilyl) ether (isomer 2)	88.9	True	$C_{21}H_{52}O_6Si_5$	174777483.1	114665453.9	72.9
10.2792	Protocatechoic acid, 3TMS derivative	88.9	True	C16H30O4Si3	27104803.8	23922438.0	193.0
10.2950	Citric acid, 4TMS derivative	92.4	True	$C_{18}H_{40}O_7Si_4$	106504159.0	119468828.1	273.0
10.3241	Citric acid, 4TMS derivative	88.1	True	$C_{18}H_{40}O_7Si_4$	142817799.3	105891386.2	273.0
10.3842	D-Fructose, 5TMS derivative	91.0	True	$C_{21}H_{52}O_6Si_5$	210686565.7	115781313.5	204.0
10.4516	D-(+)-Talofuranose, pentakis(trimethylsilyl) ether (isomer 2)	89.1	True	$C_{21}H_{52}O_6Si_5$	184561001.0	70009484.6	216.9
10.6292	D-Fructose, 1,3,4,5,6-pentakis-O-(trimethylsilyl)-, O-methyloxime	88.3	True	$C_{22}H_{55}NO_6Si_5$	128282065.2	107598635.1	307.0

10.6549	D-Fructose, 1,3,4,5,6-pentakis-O-(trimethylsilyl)-, O-methyloxime	86.8	True	$C_{22}H_{55}NO_6Si_5$	101594644.3	106586314.0	307.0
10.7737	d-Glucose, 2,3,4,5,6-pentakis-O-(trimethylsilyl)-, o-methyloxyme, (1Z)-	86.7	True	$C_{22}H_{55}NO_6Si_5$	133610277.6	113105142.3	319.0
10.7975	d-Glucose, 2,3,4,5,6-pentakis-O-(trimethylsilyl)-, o-methyloxyme, (1Z)-	87.7	True	$C_{22}H_{55}NO_6Si_5$	107547390.7	118127368.9	319.0
10.8936	D-(+)-Talose, pentakis(trimethylsilyl) ether, methyloxime (syn)	86.4	True	$C_{22}H_{55}NO_6Si_5$	181009781.2	112067712.3	319.0
10.9307	Methyl galactoside, 4TMS derivative	89.6	True	$C_{19}H_{46}O_6Si_4$	116916945.0	85558000.0	203.9
10.9615	D-Glucitol, 6TMS derivative	85.5	True	$C_{24}H_{62}O_6Si_6$	12496763.7	13930667.5	319.0
11.0103	D-Glucitol, 6TMS derivative	86.3	True	$C_{24}H_{62}O_6Si_6$	104028762.2	102536266.7	204.9
11.0106	D-Sorbitol, 6TMS derivative	85.8	True	$C_{24}H_{62}O_6Si_6$	101109025.1	100877240.3	205.0
11.0430	D-(+)-Turanose, octakis(trimethylsilyl) ether	87.4	True	$C_{36}H_{86}O_{11}Si_8$	51830744.4	44762341.2	73.0
11.0430	Maltose, octakis(trimethylsilyl) ether, methyloxime (isomer 2)	87.9	True	$C_{37}H_{89}NO_{11}Si_8$	77766977.5	52530507.1	216.9
11.1210	.beta.-D-Allopyranose, 5TMS derivative	87.9	True	$C_{21}H_{52}O_6Si_5$	166050185.2	147030437.3	203.9
11.1411	.beta.-D-Allopyranose, 5TMS derivative	86.6	True	$C_{21}H_{52}O_6Si_5$	149575034.7	147215141.3	203.9
11.3433	Palmitic Acid, TMS derivative	94.5	True	$C_{19}H_{40}O_2Si$	14475458.2	13786928.9	116.9
11.7465	Myo-Inositol, 6TMS derivative	95.1	True	$C_{24}H_{60}O_6Si_6$	146536676.7	94795723.3	72.9
13.6066	3-.alpha.-Mannobiose, octakis(trimethylsilyl) ether (isomer 1)	85.6	True	$C_{36}H_{86}O_{11}Si_8$	77102174.6	53186414.4	216.9
14.1113	Sucrose, 8TMS derivative	85.9	True	$C_{36}H_{86}O_{11}Si_8$	179173810.6	121187820.6	361.0
14.1397	D-(+)-Trehalose, octakis(trimethylsilyl) ether	85.1	True	$C_{36}H_{86}O_{11}Si_8$	409741617.4	119836756.6	361.0

Table. S3. Comparison between different biosensing assays for the detection of bacteria

Biosensing strategy	Target analyte	Linear range CFU.mL^{-1}	LOD (CFU.mL^{-1})	Advantages	Disadvantages	Ref.
Immuno magnetic separation (IMS)-PCR	*M. bovis*	10^0-10^5	57.7	High specificity	Complex procedure	[18]
IMS-LFD (lateral flow device)	*M. bovis*	10^{4-5}	1.7×10^4	novel, rapid immunochromatographic lateral flow device (LFD) as a non-invasive test to detect M. bovis cells in badger faeces	Low sensitivity	[19]
RT-PCR assay	*M. bovis*	-	10^4	High specify	DNA amplification based assay, which is time consuming and involves loads of instrumentation. Not suitable for point of care applications	[20]
Biogenic-Au nanozyme based colorimetric biosensing	*M. bovis*	10^0-10^2	~53 in washing buffer and<br>~71 in spiked sample	Low cost, effective, sensitive and specific biosensing assay	Interference of different bio matrixes in real sample analysis	This paper
Double-tagging PCR and electrochemical genosensing	*M. bovis*	-	-	Synergistic approach resulting in higher specificity	Complicated steps involved and qualitative assay	[21]
Pd@PtNPs based colorimetric biosensor	*Escherichia coli* O157:H7	-	9×10^2	Cost effective and rapid biosensing strategy	Low sensitivity	[22]
Fe_3O_4 NPC	*Listeria monocytogenes*	5.4×10^3-10^8	5.4×10^3	Low cost and highly specific biosensing strategy	Lower sensitivity	[23]

References

(1) Yildirim, A.; Mavi, A.; Kara, A. A. Determination of Antioxidant and Antimicrobial Activities of Rumex Crispus L. Extracts. *J. Agric. Food Chem.* **2001**, *49* (8), 4083–4089. https://doi.org/10.1021/jf0103572.

(2) Yang, B.; Zhao, M.; Shi, J.; Yang, N.; Jiang, Y. Effect of Ultrasonic Treatment on the Recovery and DPPH Radical Scavenging Activity of Polysaccharides from Longan Fruit Pericarp. *Food Chem.* **2008**, *106* (2), 685–690. https://doi.org/10.1016/j.foodchem.2007.06.031.

(3) Oyaizu, M. Studies on Products of Browning Reaction. Antioxidative Activities of Products of Browning Reaction Prepared from Glucosamine. *Japanese J. Nutr. Diet.* **1986**, *44* (6), 307–315. https://doi.org/10.5264/eiyogakuzashi.44.307.

(4) Jayabalan, R.; Subathradevi, P.; Marimuthu, S.; Sathishkumar, M.; Swaminathan, K. Changes in Free-Radical Scavenging Ability of Kombucha Tea during Fermentation. *Food Chem.* **2008**, *109* (1), 227–234. https://doi.org/10.1016/j.foodchem.2007.12.037.

(5) Das, B.; Moumita, S.; Ghosh, S.; Khan, M. I.; Indira, D.; Jayabalan, R.; Tripathy, S. K.; Mishra, A.; Balasubramanian, P. Biosynthesis of Magnesium Oxide (MgO) Nanoflakes by Using Leaf Extract of Bauhinia Purpurea and Evaluation of Its Antibacterial Property against Staphylococcus Aureus. *Mater. Sci. Eng. C* **2018**, *91* (September 2017), 436–444. https://doi.org/10.1016/j.msec.2018.05.059.

(6) Determinations, A.; Radical, S. F. 1811199a0. **1958**, No. 4617, 0–1.

(7) AL Waterhouse. Determination of Total Phenolics. *Curr. Protoc. Food Anal. Chem.* **2002**, *6* (1), I1.1.1-I1.1.8.

(8) Baba, S. A.; Malik, S. A. Determination of Total Phenolic and Flavonoid Content, Antimicrobial and Antioxidant Activity of a Root Extract of Arisaema Jacquemontii Blume . *J. Taibah Univ. Sci.* **2015**, *9* (4), 449–454. https://doi.org/10.1016/j.jtusci.2014.11.001.

(9) Chang, C. C.; Yang, M. H.; Wen, H. M.; Chern, J. C. Estimation of Total Flavonoid Content in Propolis by Two Complementary Colometric Methods. *J. Food Drug Anal.* **2002**, *10* (3), 178–182. https://doi.org/10.38212/2224-6614.2748.

(10) McVey, C.; Logan, N.; Thanh, N. T. K.; Elliott, C.; Cao, C. Unusual Switchable Peroxidase-Mimicking Nanozyme for the Determination of Proteolytic Biomarker. *Nano Res.* **2019**, *12* (3), 509–516. https://doi.org/10.1007/s12274-018-2241-3.

(11) Marquez, L. A.; Dunford, H. B. Mechanism of the Oxidation of 3,5,3’,5’-Tetramethylbenzidine by Myeloperoxidase Determined by Transient- and Steady-State Kinetics. *Biochemistry* **1997**, *36* (31), 9349–9355. https://doi.org/10.1021/bi970595j.

(12) Yadav, P. K.; Singh, V. K.; Chandra, S.; Bano, D.; Kumar, V.; Talat, M.; Hasan, S. H. Green Synthesis of Fluorescent Carbon Quantum Dots from Azadirachta Indica Leaves and Their Peroxidase-Mimetic Activity for the Detection of H 2 O 2 and Ascorbic Acid in Common Fresh Fruits. *ACS Biomater. Sci. Eng.* **2019**, *5* (2), 623–632. https://doi.org/10.1021/acsbiomaterials.8b01528.

(13) Kireyko, A. V.; Veselova, I. A.; Shekhovtsova, T. N. Mechanisms of Peroxidase

Oxidation of O-Dianisidine, 3,3′,5,5′- Tetramethylbenzidine, and o-Phenylenediamine in the Presence of Sodium Dodecyl Sulfate. *Russ. J. Bioorganic Chem.* **2006**, *32* (1), 71–77. https://doi.org/10.1134/S1068162006010079.

(14) Hizir, M. S.; Top, M.; Balcioglu, M.; Rana, M.; Robertson, N. M.; Shen, F.; Sheng, J.; Yigit, M. V. Multiplexed Activity of PerAuxidase: DNA-Capped AuNPs Act as Adjustable Peroxidase. *Anal. Chem.* **2016**, *88* (1), 600–605. https://doi.org/10.1021/acs.analchem.5b03926.

(15) Shah, J.; Purohit, R.; Singh, R.; Karakoti, A. S.; Singh, S. ATP-Enhanced Peroxidase-like Activity of Gold Nanoparticles. *J. Colloid Interface Sci.* **2015**, *456*, 100–107. https://doi.org/10.1016/j.jcis.2015.06.015.

(16) Deng, H. H.; Weng, S. H.; Huang, S. L.; Zhang, L. N.; Liu, A. L.; Lin, X. H.; Chen, W. Colorimetric Detection of Sulfide Based on Target-Induced Shielding against the Peroxidase-like Activity of Gold Nanoparticles. *Anal. Chim. Acta* **2014**, *852*, 218–222. https://doi.org/10.1016/j.aca.2014.09.023.

(17) Drozd, M.; Pietrzak, M.; Parzuchowski, P.; Mazurkiewicz-Pawlicka, M.; Malinowska, E. Peroxidase-like Activity of Gold Nanoparticles Stabilized by Hyperbranched Polyglycidol Derivatives over a Wide PH Range. *Nanotechnology* **2015**, *26* (49). https://doi.org/10.1088/0957-4484/26/49/495101.

(18) Stewart, L. D.; McNair, J.; McCallan, L.; Thompson, S.; Kulakov, L. A.; Grant, I. R. Production and Evaluation of Antibodies and Phage Display-Derived Peptide Ligands for Immunomagnetic Separation of Mycobacterium Bovis. *J. Clin. Microbiol.* **2012**, *50* (5), 1598–1605. https://doi.org/10.1128/JCM.05747-11.

(19) Stewart, L. D.; Tort, N.; Meakin, P.; Argudo, J. M.; Nzuma, R.; Reid, N.; Delahay, R. J.; Ashford, R.; Montgomery, W. I.; Grant, I. R. Development of a Novel Immunochromatographic Lateral Flow Assay Specific for Mycobacterium Bovis Cells and Its Application in Combination with Immunomagnetic Separation to Test Badger Faeces. *BMC Vet. Res.* **2017**, *13* (1). https://doi.org/10.1186/s12917-017-1048-x.

(20) Young, J. S.; Gormley, E.; Wellington, E. M. H. Molecular Detection of Mycobacterium Bovis and Mycobacterium Bovis BCG (Pasteur) in Soil. *Appl. Environ. Microbiol.* **2005**, *71* (4), 1946–1952. https://doi.org/10.1128/AEM.71.4.1946-1952.2005.

(21) Lermo, A.; Liébana, S.; Campoy, S.; Fabiano, S.; Inés García, M.; Soutullo, A.; Zumárraga, M. J.; Alegret, S.; Isabel Pividori, M. A Novel Strategy for Screening-out Raw Milk Contaminated with Mycobacterium Bovis on Dairy Farms by Double-Tagging PCR and Electrochemical Genosensing. *Int. Microbiol.* **2010**, *13* (2), 91–97. https://doi.org/10.2436/20.1501.01.114.

(22) Han, J.; Zhang, L.; Hu, L.; Xing, K.; Lu, X.; Huang, Y.; Zhang, J.; Lai, W.; Chen, T. Nanozyme-Based Lateral Flow Assay for the Sensitive Detection of Escherichia Coli O157:H7 in Milk. *Journal of Dairy Science*. 2018, pp 5770–5779. https://doi.org/10.3168/jds.2018-14429.

(23) Zhang, L.; Huang, R.; Liu, W.; Liu, H.; Zhou, X.; Xing, D. Rapid and Visual Detection of Listeria Monocytogenes Based on Nanoparticle Cluster Catalyzed Signal Amplification. *Biosensors and Bioelectronics*. 2016, pp 1–7.

https://doi.org/10.1016/j.bios.2016.05.100.
